# Real-world treatment patterns and outcomes for patients with extensive-stage small-cell lung cancer treated in US community oncology practices

**DOI:** 10.3389/fonc.2026.1846362

**Published:** 2026-05-22

**Authors:** Himani Aggarwal, Ke Zu, Carole R. Berini, Paul Conkling, Carlos Yugar, Jinhong Guo, Jerome Goldschmidt

**Affiliations:** 1 Merck & Co., Inc., Rahway, NJ, United States; 2 Ontada, Boston, MA, United States

**Keywords:** clinical outcomes, extensive-stage small cell lung cancer, immunotherapy, real-world evidence, treatment pattern

## Abstract

**Background:**

Extensive-stage small-cell lung cancer (ES-SCLC) is an aggressive malignancy with a poor prognosis. Advances have been made in the treatment landscape in the past decade, including integrating immune checkpoint inhibitors (ICIs) with platinum-based chemotherapy in the first-line (1L) setting and the recent approval of T-cell engagers in subsequent lines. Despite these developments, efficacy remains limited, and therapeutic resistance emerges rapidly. This study aimed to evaluate treatment patterns and clinical outcomes among real-world ES-SCLC patients treated in US community oncology practices.

**Methods:**

This retrospective cohort study used secondary structured electronic health records (EHR) data from the iKnowMed system. Adult patients diagnosed with *de novo*, or progressive/recurrent ES-SCLC who initiated 1L systemic therapy (index event) between 1 October 2018, and 30 June 2023, and had at least one follow-up encounter were identified. Patients were followed through 30 June 2024. Descriptive analyses summarized patient characteristics and treatment patterns. Real-world time on treatment (rwToT), time to next treatment (rwTTNT), and overall survival (rwOS) were assessed using Kaplan–Meier methods.

**Results:**

Among 3,484 eligible patients, 86.6% had *de novo* and 13.3% had progressive/recurrent ES-SCLC. The most common 1L treatment was ICI plus platinum-based chemotherapy (73.4%), followed by platinum-based chemotherapy only (16.5%). After ICI plus platinum-based chemotherapy, 29.6% of patients received non-platinum–based chemotherapy in 2L. Over half of patients did not receive 2L therapy (58.0%). Median rwToT, median rwTTNT, and median rwOS in 1L were 4.17 months [95% confidence interval (CI): 4.01, 4.27], 6.41 months (95% CI: 6.24, 6.54), and 9.99 months (95% CI: 9.69, 10.38), respectively. Corresponding outcomes in 2L were 2.23 months (95% CI: 2.14, 2.37), 3.61 months (95% CI: 3.35, 3.91), and 6.31 months (95% CI: 5.88, 6.87). Outcomes worsened with each subsequent line of therapy. Outcomes were generally comparable between *de novo* and progressive/recurrent ES-SCLC, although rwToT and rwTTNT in 1L were shorter for progressive/recurrent ES-SCLC.

**Conclusions:**

This study shows high utilization of ICI plus chemotherapy in 1L ES-SCLC, consistent with clinical guidelines. Despite this uptake, poor survival outcomes persisted, highlighting substantial unmet needs and the importance of developing novel treatment strategies and continuing to evaluate emerging therapies in real-world settings.

## 1 Introduction

Lung cancer is among the most commonly diagnosed cancers in the US, with over 200,000 new cases annually (1). Small-cell lung cancer (SCLC), an aggressive subtype of the disease, makes up approximately 12% of lung cancer cases (2). Over two-thirds of patients are diagnosed with the extensive stage (ES) of disease, and 5-year survival is estimated at around 6.4% (3). Even for those with limited-stage SCLC, most will have recurrence or progression within 2 years of diagnosis (4, 5).

Until 2018, platinum-based chemotherapy was mainly used in the first-line (1L) treatment of ES-SCLC. The ES-SCLC treatment landscape changed following the publication of results from the IMpower-133 trial in late September 2018, which showed that atezolizumab, in combination with carboplatin plus etoposide, demonstrated superior efficacy over carboplatin plus etoposide as a 1L treatment of ES-SCLC (6). This regimen was later approved in this indication on 18 March 2019 (7) and became a preferred treatment option (8). In March 2020, durvalumab in combination with carboplatin or cisplatin and etoposide was approved as a 1L treatment for ES-SCLC based on the results from the CASPIAN study (9, 10). As such, durvalumab in combination with platinum-based chemotherapy is also a preferred 1L regimen option. Subsequent therapies include traditional chemotherapies, such as irinotecan and topotecan, as well as rechallenge with platinum-based therapy and recently approved novel agents, including lurbinectedin and tarlatamab (11).

For patients with limited−stage SCLC (LS−SCLC), the standard of care includes concurrent platinum–etoposide chemotherapy with radiotherapy (CCRT) (3). Major advancements have recently emerged through the phase III ADRIATIC trial, which evaluated durvalumab as consolidation therapy in patients who had not progressed following CCRT and demonstrated significant improvements in both overall survival and progression−free survival. These results established maintenance therapy with durvalumab post-CCRT as a new standard of care for LS−SCLC (12). Despite aggressive multimodal therapy, it is estimated that 60% of LS−SCLC patients recur or progress within 3 months after 1L (3).

While clinical trials have expanded the therapeutic options for ES-SCLC, real-world evidence remains sparse, with limited information on the real-world treatment patterns and clinical outcomes in this patient population (11, 13). Understanding current real-world patient outcomes will enable the identification of patients’ unmet needs in their treatment journey and help inform future clinical development (14). This study aimed to address this gap and contribute to the body of real-world evidence, characterizing the treatment patterns and clinical outcomes for ES-SCLC patients treated in US community oncology settings.

## 2 Methods

### 2.1 Study design and setting

This was a retrospective cohort study utilizing secondary electronic health record (EHR) data from The US Oncology Network and non-network practices using the iKnowMed (iKM) EHR system, which is specifically designed for oncology outpatient practices. Data were collected from structured fields via programmatic queries of the iKM EHR. The protocol for this study received Castle institutional review board (IRB), IORG0010151/IRB00012054, approval in August 2024 (Case #: 17941.3).

### 2.2 Study population

Adult patients (aged ≥ 18 years) with ES-SCLC who had initiated 1L systemic treatment between 1 October 2018 and 30 June 2023 were eligible for inclusion in the study. Patients had to have at least 1 additional clinical encounter after treatment initiation to demonstrate continuity of care. Eligible patients included (1) *de-novo* ES-SCLC, for those initially diagnosed with stage IV or *de-novo* metastasis; and (2) progressive/recurrent ES-SCLC, for those initially diagnosed with stages I–III who subsequently progressed or recurred to ES-SCLC (i.e., had evidence of metastatic disease). Evidence of metastatic disease was defined as having metastases based on TNM staging documentation, documentation of metastatic site location, subsequent documentation of stage IV after stages I–III diagnosis (limited stage), or having received at least two systemic therapies after limited-stage (stages I–III) diagnosis. Date of ES-SCLC diagnosis was defined as the first date of *de-novo* stage IV or *de-novo* metastasis (M>0 or documentation of metastatic site) for the *de-novo* ES-SCLC subgroup and the first date of metastatic evidence after initial stages I–III diagnosis (date of TNM stage with M1 or greater, date of stage IV, or date of second systemic treatment initiation) for the progressive/recurrent ES-SCLC subgroup. The qualifying 1L in extensive stage setting was defined as the first regimen initiated within 14 days prior to or 30 days following the diagnosis of ES-SCLC. Patients enrolled in interventional clinical trials or receiving treatment indicated for another primary cancer were excluded. All patients were followed through 30 June 2024.

### 2.3 Treatment regimen and line of therapy

The index date was defined as the date of initiation of 1L systemic treatment for ES-SCLC, as described above. If a regimen consisted of more than one drug, with drugs given on different dates, the date of the administration of the first drug in the regimen was used. Treatment sequences were defined based on the absolute order, using start and stop dates as recorded in the EHR structured fields. All anticancer drugs with a start date that occurred within 28 days of the start of the first anticancer drug were combined into one regimen. If there was a gap of 120 days or more in cancer drug administration or if a new cancer drug was introduced past the 28-day threshold, the regimen was advanced to the subsequent line of therapy (LOT). Maintenance immunotherapy with atezolizumab or durvalumab following initial platinum-etoposide plus ICI combination was consolidated within the same line of therapy and not considered a separate regimen.

### 2.4 Real-world clinical outcomes

Real-world time on treatment (rwToT) was defined as the interval between the start of a LOT and its discontinuation or the date of death due to any cause if it occurred first. Patients who did not discontinue treatment and had no record of death on or before the end of the study observation period were censored on the last contact date available in the dataset.

Real-world time to next treatment (rwTTNT) was defined as the interval between the initiation of a LOT and the initiation of a subsequent LOT, or the date of death due to any cause if it occurred first. Patients who did not initiate a subsequent LOT and had no record of death on or before the end of the study observation period were censored on the last contact date available in the dataset.

Real-world overall survival (rwOS) was defined as the interval between the start of a LOT and the date of death due to any cause. Patients who did not die on or before the end of the study observation period were censored on the last contact date available in the dataset.

### 2.5 Statistical analysis

Descriptive statistics were used to summarize demographic and clinical characteristics, as well as treatment sequences from 1L to the third line of therapy (3L). Sankey diagrams were created to visualize the treatment sequences from 1L to 3L. The Kaplan-Meier method was used to analyze time-to-event outcomes within each LOT. All analyses were performed in SAS software (SAS Institute Inc., Cary, NC, USA).

## 3 Results

**Figure 1:**
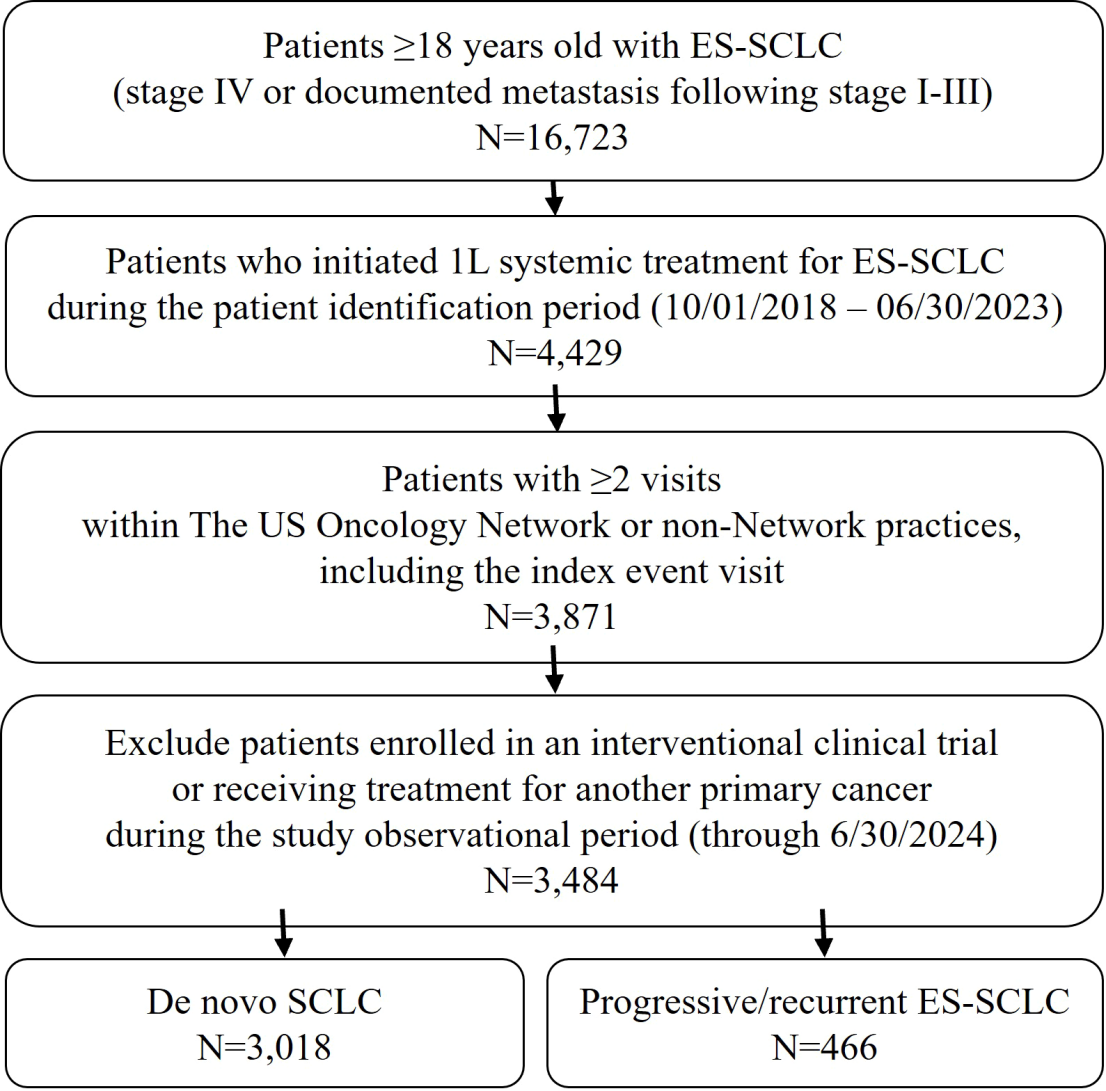
Patient attrition. 1L, first-line; ES-SCLC, extensive-stage small-cell lung cancer; SCLC, small-cell lung cancer; US, United States.

A total of 3,484 eligible patients were included in this study, of whom 3,018 and 466 were *de novo* and progress/recurrent ES-SCLC, respectively (**Figure 1**). The median follow-up for the overall population was 8.6 months (IQR: 4.6, 14.6). Overall, the median time from ES-SCLC diagnosis to initiation of 1L systemic treatment was 7 days (IQR: 1, 16).

### 3.1 Demographic and clinical characteristics

In the overall study population, the median age was 68.3 years (interquartile range [IQR]: 62.2, 74.5), with more than 80% of patients being over 60 years old. The proportion of male and female patients was similar, 52.1% female and 47.9% male. Most patients were White (73.6%), non-Hispanic (80.1%), and treated at practices in the South (40.9%), Midwest (32.7%), and West (22.7%). Demographic characteristics were similar between the *de novo* and the progressive/recurrent subgroups (**Table 1**).

**Table 1:** Demographic characteristics of ES-SCLC patients.

Variable	Overall ES-SCLC (*n* = 3, 484)	*De-novo* ES-SCLC (*n* = 3, 018)	Progressive/recurrent ES-SCLC (*n* = 466)
Age at index (years)			
*N*	3484	3018	466
Mean (*SD*)	68.21 (8.82)	68.25 (8.68)	67.92 (9.70)
Median	68.3	68.36	67.97
IQR	62.17, 74.47	62.29, 74.43	61.63, 74.76
Min, Max	29.52, 90+	31.42, 90+	29.52, 90+
Age group at index (years) – *n* (%)			
18–49	65 (2.0)	52 (1.9)	13 (3.1)
50–59	430 (13.4)	369 (13.2)	61 (14.3)
60–69	1231 (38.2)	1077 (38.5)	154 (36.2)
≥ 70	1494 (46.4)	1296 (46.4)	198 (46.5)
Sex – *n* (%)			
Female	1816 (52.1)	1540 (51.0)	276 (59.2)
Male	1668 (47.9)	1478 (49.0)	190 (40.8)
Race – *n* (%)			
American Indian or Alaska Native	19 (0.5)	17 (0.6)	<3
Asian	32 (0.9)	26 (0.9)	6 (1.3)
Black or African American	196 (5.6)	165 (5.5)	31 (6.7)
White	2563 (73.6)	2197 (72.8)	366 (78.5)
Other	79 (2.3)	74 (2.5)	5 (1.1)
Not documented	595 (17.1)	539 (17.9)	56 (12.0)
Ethnicity – *n* (%)			
Hispanic or Latino	82 (2.4)	68 (2.3)	14 (3.0)
Not Hispanic or Latino	2790 (80.1)	2397 (79.4)	393 (84.3)
Not documented	612 (17.6)	553 (18.3)	59 (12.7)
Practice region at index – *n* (%)			
Midwest	1141 (32.7)	985 (32.6)	156 (33.5)
Northeast	127 (3.6)	108 (3.6)	19 (4.1)
South	1426 (40.9)	1231 (40.8)	195 (41.8)
West	790 (22.7)	694 (23.0)	96 (20.6)
Not documented	0	0	0

ES-SCLC, extensive-stage small-cell lung cancer; IQR, interquartile range; SCLC, small-cell lung cancer; SD, standard deviation.

Eastern Cooperative Oncology Group (ECOG) scores were documented for over 90% of the patient population, with 73.4% having a score of 0 or 1. The majority of patients had a median body mass index (BMI) of 26 (IQR: 22.6, 30.3). Among patients with documentation, most were current or former tobacco users (95%). Laboratory values and TNM stage were not well documented across the study population (>50%). Likewise, metastatic sites and comorbidities were also frequently not documented (>60%), and it was not possible to distinguish the true absence of the condition from undocumented information. The median time from initial diagnosis to initiation of 1L systemic treatment was 9 days and 256 days for the *de-novo* ES-SCLC and progressive/recurrent SCLC subgroups, respectively. Other clinical characteristics were similar in both subgroups (**Table 2**).

**Table 2:** Clinical characteristics of ES-SCLC patients.

Analysis variable	Overall ES-SCLC (*n* = 3, 484)	*De-novo* ES-SCLC (*n* = 3, 018)	Progressive/recurrent ES-SCLC (*n* = 466)
Follow-up duration (months)			
*N*	3, 484	3, 018	466
Mean (SD)	11.82 (11.36)	11.58 (10.85)	13.36 (14.16)
Median	8.61	8.71	8.10
IQR	4.60, 14.55	4.63, 14.19	4.29, 16.80
Min, Max	0.03, 74.22	0.03, 69.39	0.10, 74.22
Time from initial SCLC diagnosis (stages I–III) to ES-SCLC diagnosis (days)			
*N*	-	–	466
Mean (SD)	–	–	319.41 (363.23)
Median	–	–	256
IQR	–	–	98.75, 404.25
Min, Max	–	–	1.00, 3, 435.00
Time from ES-SCLC diagnosis to index date (days)			
*N*	3, 484	3018	466**
Mean (SD)	9.27 (9.27)	10.62 (9.01)	–
Median	7	9	–
IQR	1.00, 16.00	4.00, 18.00	–
Min, Max	−14.00, 30.00	−14.00, 30.00	–
Time from initial SCLC diagnosis (stages I–III) to index date (days)			
*N*	-	-	466
Mean (SD)	–	–	320.0 (363.2)
Median	-	–	256
IQR	-	–	102.0, 405.0
Min, Max	-	–	0, 3, 435
Sites of metastases at metastatic documentation (± 120 days) – *n* (%)			
Liver	427 (12.3)	416 (13.8)	11 (2.4)
Bone	300 (8.6)	284 (9.4)	16 (3.4)
Brain	205 (5.9)	194 (6.4)	11 (2.4)
Adrenal Gland	135 (3.9)	134 (4.4)	1 (0.2)
Lung	103 (3.0)	97 (3.2)	6 (1.3)
Pleura	60 (1.7)	55 (1.8)	5 (1.1)
Mediastinum	56 (1.6)	54 (1.8)	< 3
Other	819 (23.5)	757 (25.1)	62 (13.3)
Retroperitoneum	13 (0.4)	13 (0.4)	0 (0.0)
Pancreas	13 (0.4)	12 (0.4)	< 3
Peritoneum	15 (0.4)	13 (0.4)	< 3
Bronchus	16 (0.5)	14 (0.5)	< 3
Not documented	2111 (60.6)	1737 (57.6)	374 (80.3)
Count of metastatic site(s) at metastatic documentation (± 120 days) – *n* (%)			
1	897 (25.7)	823 (27.3)	74 (15.9)
2	282 (8.1)	270 (8.9)	12 (2.6)
3	116 (3.3)	112 (3.7)	4 (0.9)
4+	78 (2.2)	76 (2.5)	< 3
None/Not documented*	2111 (60.6)	1737 (57.6)	374 (80.3)
Stage at initial SCLC diagnosis – *n* (%)			
Stage I	35 (1.0)	0 (0.0)	35 (7.5)
Stage II	85 (2.4)	0 (0.0)	85 (18.2)
Stage III	346 (9.9)	0 (0.0)	346 (74.2)
Stage IV	3018 (86.6)	3018 (100.0)	0 (0.0)
Not documented	< 3	0 (0.0)	< 3
Weight (kg) at baseline			
Patients with available data	3424	2967	457
Mean (SD)	76.66 (19.32)	76.89 (19.33)	75.16 (19.21)
Median	74.5	74.84	72.57
IQR	62.60, 88.00	63.05, 88.63	61.69, 85.28
Min, Max	22.68, 175.99	22.68, 175.99	34.47, 150.14
Body mass index (BMI, kg/m^2^) at baseline			
Patients with available data	3374	2, 931	443
Mean (SD)	26.91 (6.00)	26.94 (6.01)	26.74 (5.90)
Median	26.15	26.18	25.77
IQR	22.63, 30.26	22.63, 30.41	22.66, 29.69
Min, Max	15.62, 50.75	15.62, 50.75	15.70, 49.99
BMI categories at baseline – *n* (%)			
Underweight (< 18.5)	179 (5.2)	158 (5.3)	21 (4.5)
Normal (18.5 ≤ BMI < 25)	1, 234 (35.7)	1, 059 (35.4)	175 (37.9)
Overweight (25 ≤ BMI < 30)	1, 069 (30.9)	922 (30.8)	147 (31.8)
Obese (BMI ≥ 30)	892 (25.8)	792 (26.5)	100 (21.6)
Not documented	80 (2.3)	61 (2.0)	19 (4.1)
Tobacco use at baseline – *n* (%)			
No history of tobacco use	97 (2.8)	91 (3.0)	6 (1.3)
Current tobacco use	743 (21.3)	698 (23.1)	45 (9.7)
Former tobacco use	1, 259 (36.1)	1, 204 (39.9)	55 (11.8)
Not documented	1, 385 (39.8)	1, 025 (34.0)	360 (77.3)
ECOG performance score at baseline – *n* (%)			
0	655 (18.8)	556 (18.4)	99 (21.2)
1	1, 902 (54.6)	1, 648 (54.6)	254 (54.5)
2	560 (16.1)	491 (16.3)	69 (14.8)
≥ 3	54 (1.5)	47 (1.6)	7 (1.5)
Not documented	313 (9.0)	276 (9.1)	37 (7.9)
Deyo-Charlson Comorbidities documented 6 months prior to and including index - *n* (%)			
Chronic pulmonary disease	391 (11.2)	350 (11.6)	41 (8.8)
Diabetes	245 (7.0)	224 (7.4)	21 (4.5)
Rheumatologic disease/connective tissue disease	34 (1.0)	31 (1.0)	3 (0.6)
Renal disease	62 (1.8)	56 (1.9)	6 (1.3)
Cerebrovascular disease	35 (1.0)	26 (0.9)	9 (1.9)
Congestive heart failure	42 (1.2)	37 (1.2)	5 (1.1)
Peripheral vascular disease/Bypass	31 (0.9)	27 (0.9)	4 (0.9)
Mild liver disease	20 (0.6)	18 (0.6)	< 3
Moderate-severe liver disease	< 3	0 (0.0)	< 3
Diabetes with sequelae	7 (0.2)	6 (0.2)	< 3
Dementia/Alzheimer’s	5 (0.1)	3 (0.1)	< 3
Acute myocardial infarction (MI) or history of MI	27 (0.8)	25 (0.8)	< 3
HIV/AIDS	< 3	< 3	0 (0.0)
Paralysis	3 (0.1)	< 3	< 3
Peptic or gastric ulcer disease	6 (0.2)	4 (0.1)	< 3
No/Not documented	2, 800 (80.4)	2, 408 (79.8)	392 (84.1)
Deyo-Charlson Comorbidity Index documented 6 months prior to and including index - *n* (%)			
0*	2, 800 (80.4)	2, 408 (79.8)	392 (84.1)
1–2	623 (17.9)	555 (18.4)	68 (14.6)
3–4	56 (1.6)	50 (1.7)	6 (1.3)
≥ 5	5 (0.1)	5 (0.2)	0 (0.0)
Lactate dehydrogenase (LDH) at baseline (U/L)- *n* (%)			
Patients with available data n(%)	1, 012 (29.0)	879 (29.1)	133 (28.5)
Mean (SD)	455.82 (586.95)	484.03 (621.88)	269.40 (161.07)
Median	288	303	208.54
IQR	203.78, 488.50	209.00, 518.00	168.00, 311.00
Min, Max	72.00, 10, 610.00	72.00, 10610.00	86.00, 991.00
Lactate dehydrogenase (LDH) at baseline - *n* (%)			
Low (< 140 U/L)	43 (1.2)	31 (1.0)	12 (2.6)
Normal (140–280 U/L)	445 (12.8)	366 (12.1)	79 (17.0)
Elevated (> 280 U/L)	524 (15.0)	482 (16.0)	42 (9.0)
Not documented	2, 472 (71.0)	2, 139 (70.9)	333 (71.5)

AIDS, acquired immunodeficiency syndrome; BMI, body mass index; ECOG, Eastern Cooperative Oncology Group; ES-SCLC, extensive-stage small-cell lung cancer; HIV, human immunodeficiency virus; IQR, interquartile range; LDH, lactate dehydrogenase; LOT, line of therapy; MI, myocardial infarction; SCLC, small-cell lung cancer; SD, standard deviation; TNM, tumor-node-metastasis staging.

*As it is not possible to distinguish between no comorbidly versus the absence of documentation in the EHR data, the proportion of patients with a comorbidity index of 0 is likely inflated.

**Since most patients in this cohort were identified based on having received at least two systemic therapies after limited-stage (stages I–III) diagnosis, time from initial SCLC diagnosis (stages I–III) to ES-SCLC diagnosis (days) and time from initial SCLC diagnosis (stages I–III) to index date (days) are presented instead, as they are more informative.

### 3.2 Treatment patterns/distribution

**Figure 2:**
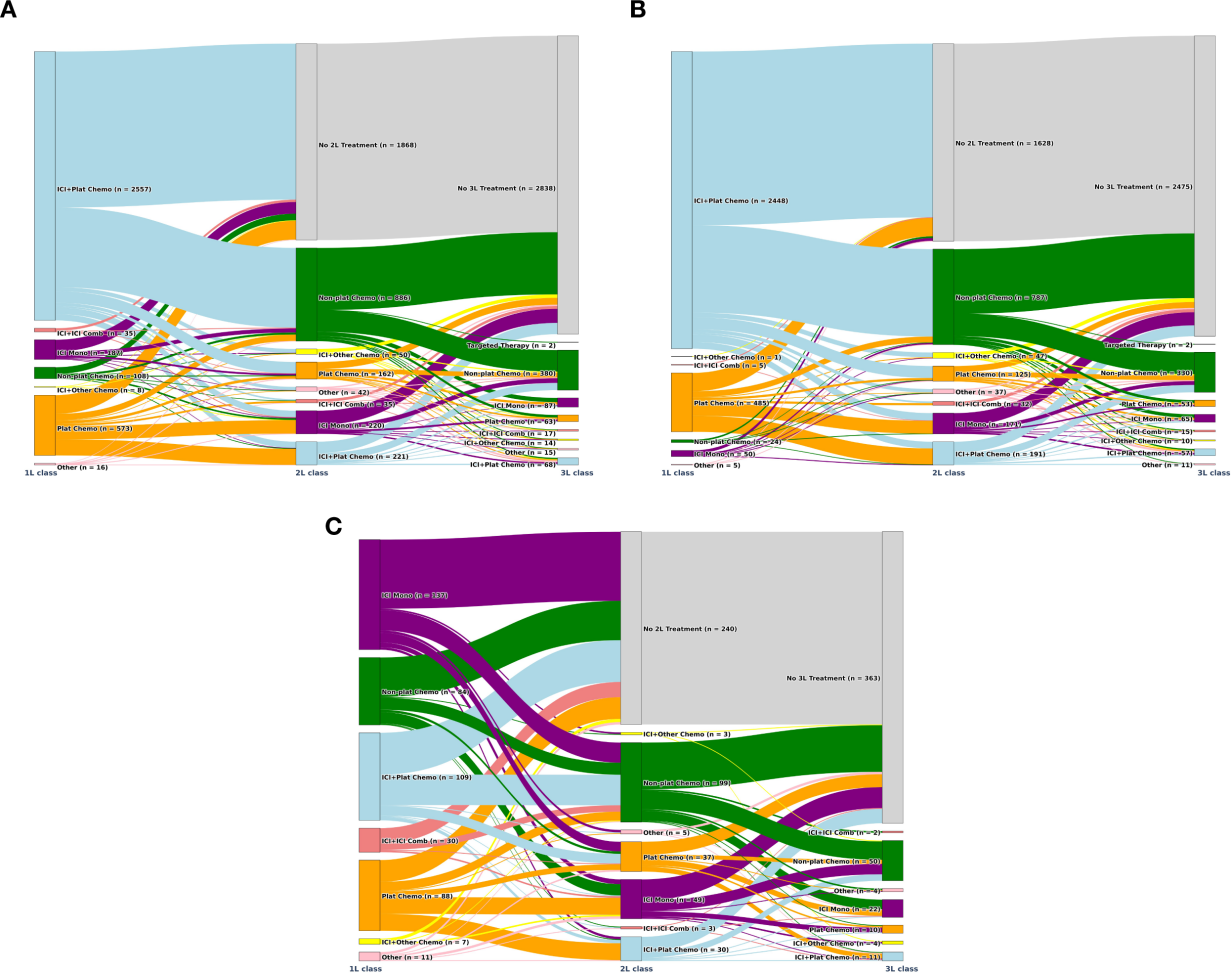
Sankey diagrams for 1L to 3L treatment for **(A)** the overall ES-SCLC population, **(B)** the de novo ES-SCLC population, **(C)** the progressive/recurrent ES-SCLC population.

Overall, the most common treatment in 1L was ICI plus platinum-based chemotherapy (73.4%), followed by platinum-based chemotherapy alone (16.5%). Other regimens were ICI monotherapy (5.4%) and non-platinum–based chemotherapy (3.1%). Subsequent to ICI plus platinum-based chemotherapy, 29.6% of patients went on to receive non-platinum–based chemotherapy. Nonetheless, over half of patients did not receive 2L therapy (58.0%). These treatment patterns in the overall population were driven by the *de novo* patients subgroup due to the considerably larger sample size. Treatments in the progressive/recurrent subgroup were more heterogeneous, with the main regimen class in 1 L being ICI monotherapy (29.4%), followed by ICI plus platinum-based chemotherapy (23.4%), platinum-based chemotherapy (18.9%), and non-platinum–based chemotherapy alone (18.0%). After ICI monotherapy, 18.3% of patients received non-platinum–based chemotherapy in 2L; however, the majority of patients in this subgroup were not observed to advance to 2L (62.8%; **Figure 2**; **Supplementary Table 1**).

### 3.3 Real-world clinical outcomes

Real-world clinical outcomes are presented in **Table 3**.

**Table 3:** Real-world clinical outcome for ES-SCLC patients.

LOT	Overall ES-SCLC		*De-novo* ES-SCLC		Progressive/recurrent ES-SCLC	
	*N*	Median (95% CI)	*N*	Median (95% CI)	*N*	Median (95% CI)
rwToT (months)						
**1L**	3, 484	4.17 (4.01, 4.27)	3, 018	4.30 (4.17, 4.44)	466	2.86 (2.50, 3.25)
**2L**	1, 616	2.23 (2.14, 2.37)	1, 390	2.23 (2.14, 2.37)	226	2.17 (1.64, 2.79)
**3L**	646	1.87 (1.64, 2.10)	543	2.00 (1.77, 2.20)	103	1.05 (0.72, 1.61)
rwTTNT (months)						
**1L**	3, 484	6.41 (6.24, 6.54)	3, 018	6.47 (6.31, 6.64)	466	5.59 (4.76, 6.24)
**2L**	1, 616	3.61 (3.35, 3.91)	1, 390	3.58 (3.35, 3.91)	226	3.71 (2.92, 4.53)
**3L**	646	2.92 (2.76, 3.25)	543	3.06 (2.79, 3.48)	103	2.37 (1.97, 3.02)
rwOS (months)						
**1L**	3, 484	9.99 (9.69, 10.38)	3, 018	9.99 (9.69, 10.38)	466	10.22 (8.64, 12.39)
**2L**	1, 616	6.31 (5.88, 6.87)	1, 390	6.08 (5.68, 6.64)	226	8.02 (6.34, 9.53)
**3L**	646	5.82 (5.26, 6.28)	543	5.68 (5.19, 6.21)	103	6.31 (4.37, 9.36)

1L, first-line; 2L, second-line; 3L, third-line; CI, confidence interval; ES-SCLC, extensive-stage small-cell lung cancer; LOT, line of therapy; rwOS, real-world overall survival; rwToT, real-world time on treatment; rwTTNT, real-world time to next treatment.

Overall, median rwToT was 4.17 months (95% CI: 4.01, 4.27) in the 1 L setting. Median rwToT decreased with each advancement in LOT, 2.23 months (95% CI: 2.14, 2.37) and 1.87 months (95% CI: 1.64, 2.10) in the 2L and 3L settings, respectively. It was lower in the progressive/recurrent subgroup compared to the *de novo* subgroup, with non-overlapping confidence intervals, 2.86 months (95% CI: 2.50, 3.25) versus 4.30 months (95% CI: 4.17, 4.44), respectively, for 1 L therapy. This difference was not observed in the 2L setting, with both subgroups having a median treatment duration of approximately 2 months. In the 3L setting, median rwToT was 1.05 months (95% CI: 0.72, 1.61) in the recurrent progressive group compared to 2.00 months (95% CI: 1.77, 2.20) in the *de novo* group.

The median 1 L rwTTNT was 6.41 months (95% CI: 6.24, 6.54) overall and decreased with each advancement in LOT, to 3.61 months (3.35, 3.91) and 2.92 months (2.76, 3.25) in the 2L and 3L settings, respectively. The median TTNT was lower in the progressive/recurrent subgroup compared to the *de novo* subgroup, with non-overlapping confidence intervals, 5.59 months (95% CI: 4.76, 6.24) versus 6.47 months (95% CI: 6.31, 6.64), respectively, in the 1L setting. This difference between subgroups was not observed in subsequent LOTs.

The median rwOS was 9.99 months (95% CI: 9.69, 10.38) overall for 1L and decreased with each advancement in LOT, 6.31 months (5.88, 6.87) and 5.82 months (5.26, 6.28) for 2L and 3L, respectively. It was slightly higher in the progressive/recurrent subgroup compared to the *de-novo* subgroup, although the difference was not significant, with overlapping confidence intervals.

## 4 Discussion

This real-world study demonstrates that, despite therapeutic advancement for patients with ES-SCLC, clinical outcomes for patients with *de novo* or progressive/recurrent disease remain uniformly poor across 1L–3L regimens. These findings underscore persistent challenges in managing ES-SCLC despite the introduction of ICIs. As other novel therapies are developed, there is an opportunity to tailor treatment strategies for each patient subgroup.

Contemporary real-world evidence on the treatment landscape in ES-SCLC is limited in the US. A previous study by Zu et al. (2023) used the iKM EHR to identify ES-SCLC patients treated in the US community oncology setting from 1 October 2018 to 29 February 2020, where over a third of patients with *de novo* ES-SCLC did not receive an ICI-containing regimen in the 1L setting (15). The current study, which expands on Zu et al., includes more patients and longer follow-up, and it indicates higher utilization of ICI plus platinum-based chemotherapy in real-world practice. This has been confirmed in another recent US-based real-world study, in which it was observed that over 70% of ES-SCLC patients received ICI-containing regimen in the 1L setting (16). While most patients were not observed to receive treatment after 1L, among those who received subsequent therapy, there was more heterogeneity in treatment pathways. This is consistent with observations from two recent studies by Sankar et al. (2025). One focused on ES-SCLC treatment after 1L platinum-based chemotherapy ± ICI and noticed inconsistent treatments beyond 1L (17). The other examined healthcare resource utilization in ES-SCLC patients from US claims-based data and observed high variability in treatment regimens in 2L+, concluding a lack of standard of care in subsequent LOTs (18). The median time to next treatment was in line with other recently published real-world studies. For instance, Ganti et al. (2024) observed a median duration of immunotherapy (including maintenance) of 4.9 months (IQR: 2.8, 7.10) and a median rwTTNT of 6.7 months (95% CI: 6.5, 6.9) in 1L (19). Another study of US patients with ES-SCLC reported a median rwTTNT of about 6 months in 1L and about 4–5 months in 2L (16). Both studies examined patients who received an ICI-containing regimen. Few real-world studies directly compared progressive/recurrent to *de novo* patients, and none reported differences in rwTTNT or rwToT by those specific subgroups (20, 21). A well-known factor contributing to uniformly poor outcomes across LOT is the rapid emergence of treatment resistance in ES-SCLC patients. Understanding the mechanisms underlying primary and acquired resistance is an active area of research, and advances in precision medicine offer hope for more effective treatment strategies (24, 25).

The survival outcome observed in the current study aligns with prior real-world evidence. For example, another study of EHR data from US patients with ES-SCLC treated between 2013 and 2021 had a median OS of about 8 months in 1L and 4–5 months in 2L (22). A study using Veteran Affairs data observed a median OS of 9 months among patients treated with ICI and chemotherapy between 2020 and 2023 (23). This is lower than what has been observed in recent clinical trials, in which median OS reached 13 months (3). For instance, in the IMpower133 trial, patients receiving atezolizumab plus carboplatin and etoposide in 1L had a median OS of 12.3 months (6), and in the CASPIAN trial, patients receiving durvalumab plus carboplatin/cisplatin and etoposide in 1L had a median OS of 13.0 months (10). Results from real-world studies are not expected to align perfectly with results from clinical trials due to known differences in populations. For instance, real-world patient populations tend to be older with poor performance status and higher comorbidity burden, compared to clinical trial participants. Therefore, it is expected that survival outcomes based on real-world data are inferior compared to those observed in clinical trials. Nonetheless, despite the increasing use of ICIs, this study highlights the unmet needs of ES-SCLC patients in the real world.

In May 2024, the FDA granted accelerated approval to tarlatamab for the treatment of ES-SCLC in 2L+, which was converted into full approval in November 2025 (26, 27). This was based on the results from the DeLLphi-304 trial, which demonstrated a median OS of 13.6 months (95% CI: 11.1, not reached) (28). In October 2025, the FDA approved lurbinectedin in combination with atezolizumab for use as a maintenance therapy after chemo-immunotherapy (29). This was based on the IMforte trial, resulting in a median OS of 13.2 months (95% CI: 11.9, 16.4) (30). The approvals of these therapies are too recent to be reflected in the current study. Given the promising clinical trial results, the emergence of new therapies offers hope for the treatment of ES-SCLC patients. Further investigation is needed to evaluate the integration of tarlatamab and the combination of lurbinectedin and atezolizumab into clinical practice and confirm their survival benefits in real-world settings. Furthermore, the current study highlighted the severe attrition from 1L to subsequent lines of therapy. To address this, novel therapies should be investigated in 1L to extend their potential benefits to the majority of patients.

While this study provides valuable insights into real-world treatment patterns and outcomes for patients with ES-SCLC, its limitations must be acknowledged. For this study, data were sourced exclusively from structured fields within the EHR system. EHR data are not collected for research purposes, and as such, some variables of interest were inconsistently documented or were subject to misclassification. For example, there was a high rate of undocumented sites of metastases, which can be an important factor in patient prognosis. LOT was defined using a treatment-based algorithm and therefore may not consistently reflect disease progression. Additionally, in the absence of other evidence, patients with progressive/recurrent ES-SCLC were defined as those who had received at least two systemic therapies after a diagnosis of LS-SCLC. While this approach potentially introduced misclassification of progressive/recurrent ES-SCLC, it is expected that few, if any, LS-SCLC cases were included as progressive/recurrent ES-SCLC. This approach was chosen based on the clinical management of LS-SCLC as an adequate treatment-based algorithm used to identify most patients in the progressive/recurrent ES-SCLC group, since a subsequent systemic treatment is indicative of progression or recurrence, accounting for 1L treatment with CCRT for LS-SCLC. Finally, while it was expected that only about a quarter of ES-SCLC cases would be in the progressive/recurrent subgroup (21), this group was limited by its small sample size.

### 4.1 Conclusion

This study contributes to the body of real-world evidence in the treatment landscape and outcomes of patients with ES-SCLC in a representative sample of the US population treated in community oncology settings. Results highlight that clinical outcomes for ES-SCLC patients are suboptimal and that opportunities exist to address the unmet needs of patients by optimizing current regimens, identifying biomarkers that refine patient selection, and developing novel agents capable of producing deeper and more durable responses. Ongoing clinical research is increasingly focused on rational combinations of immunotherapy, targeted modalities, and next-generation biologics to further improve outcomes in this historically difficult-to-treat disease. As new therapy options obtain FDA approval and are included in clinical guideline recommendations, there is a need for additional real-world studies to provide evidence that informs clinical development and practice.

## Data Availability

The data utilized in this retrospective study are derived from patient records and are subject to HIPAA regulations to ensure the privacy and protection of personal health information. The study was conducted under a waiver of authorization granted by the Institutional Review Board (IRB) which limits our ability to disclose such sensitive information publicly. Requests to access these datasets should be directed to OntadaExpert@McKesson.com.
