# Supplementary material for: Real-world treatment patterns and outcomes for patients with extensive-stage small-cell lung cancer treated in US community oncology practices
Source: Front Oncol. 2026 May 22;16:1846362. doi: 10.3389/fonc.2026.1846362 (PMC13236545; doi:10.3389/fonc.2026.1846362)
Supplement: Supplementary file 1 [file SupplementaryFile1.docx]

Supplementary Material

# Supplementary Figures

**Supplementary Figure 1.** Kaplan-Meier curves for real-world time on treatment (rwToT), by ES-SCLC type, in A) first-line, B) second-line, and C) third-line therapy.

A)


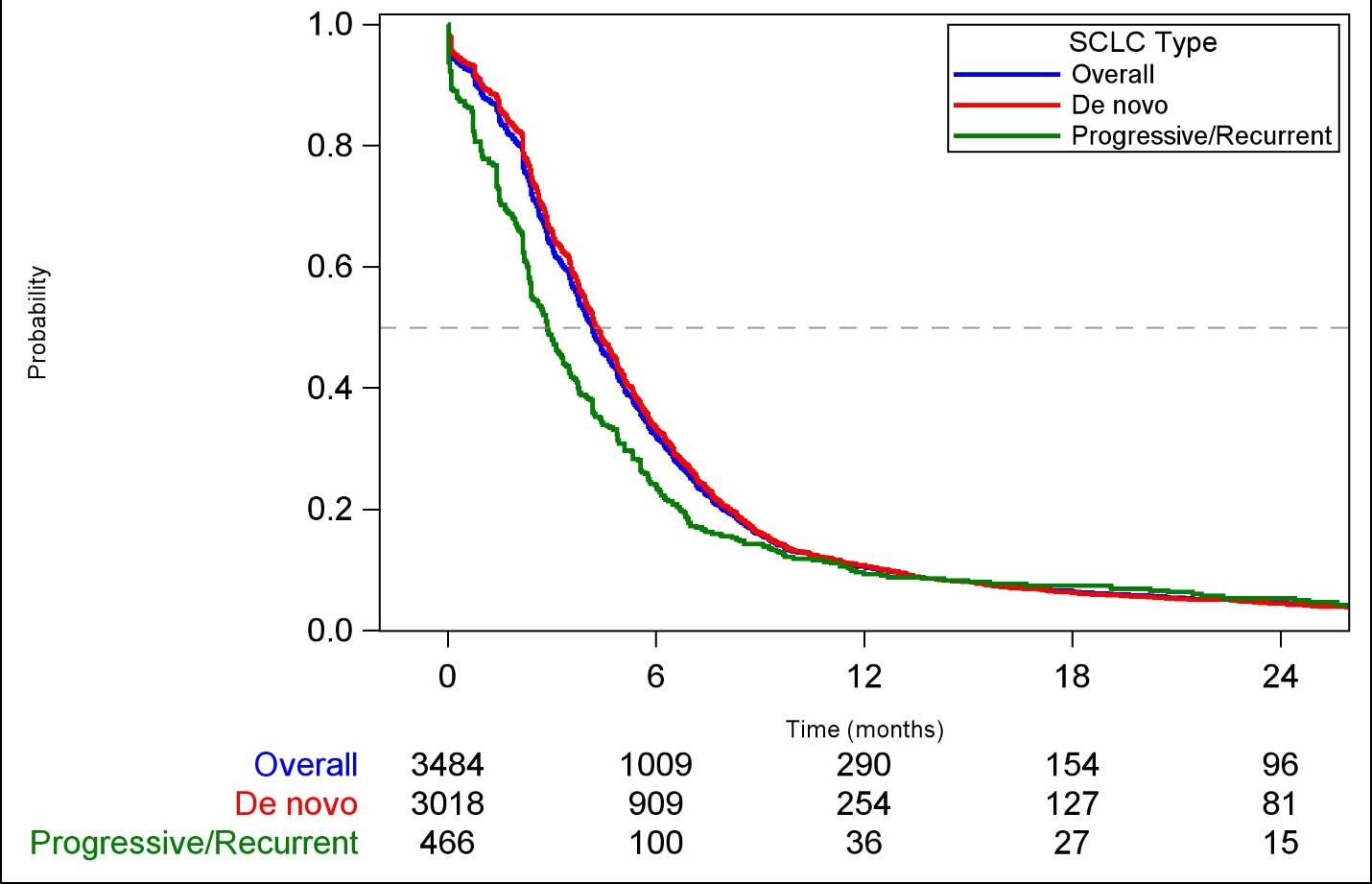


B)


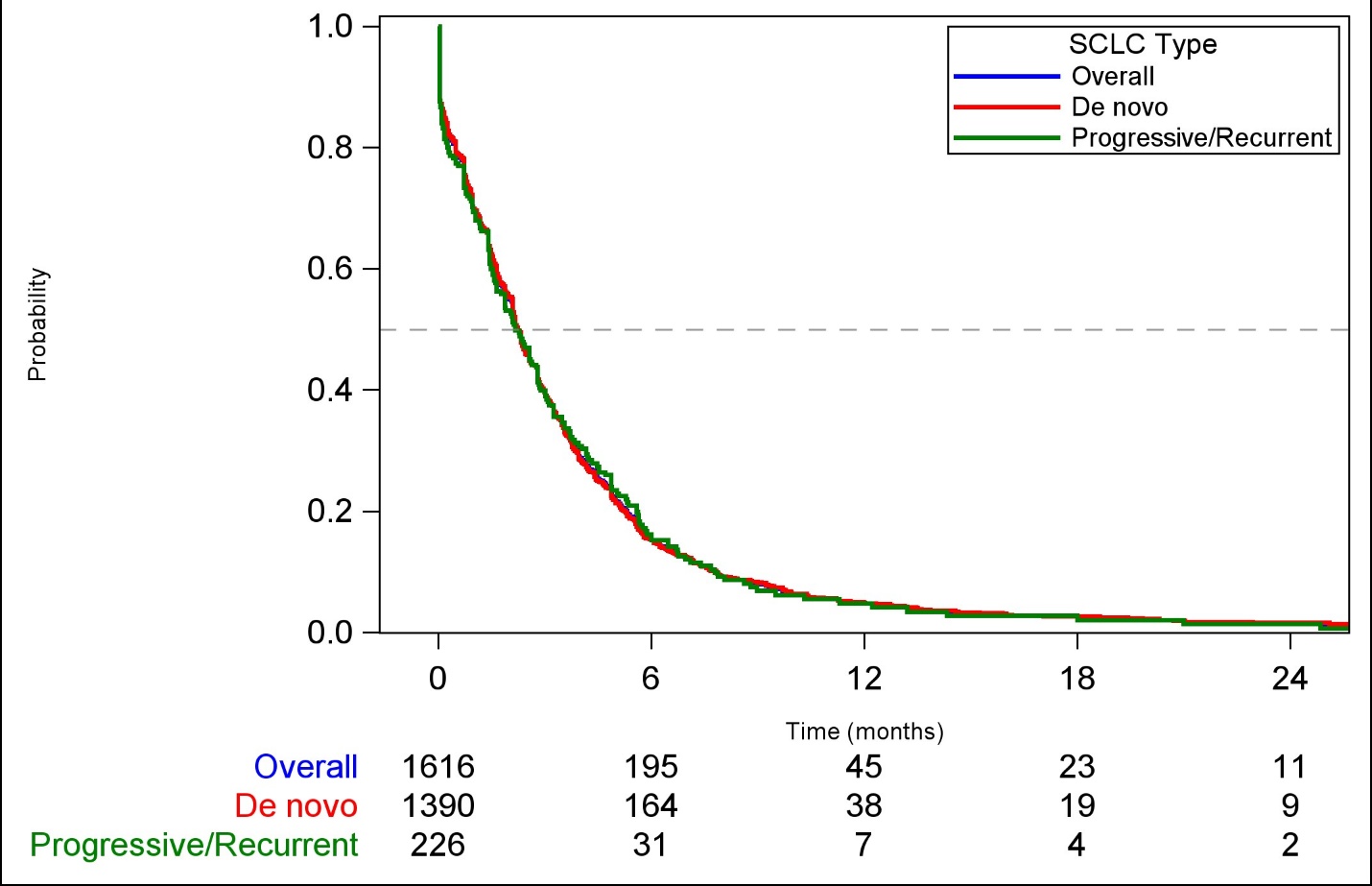


C)


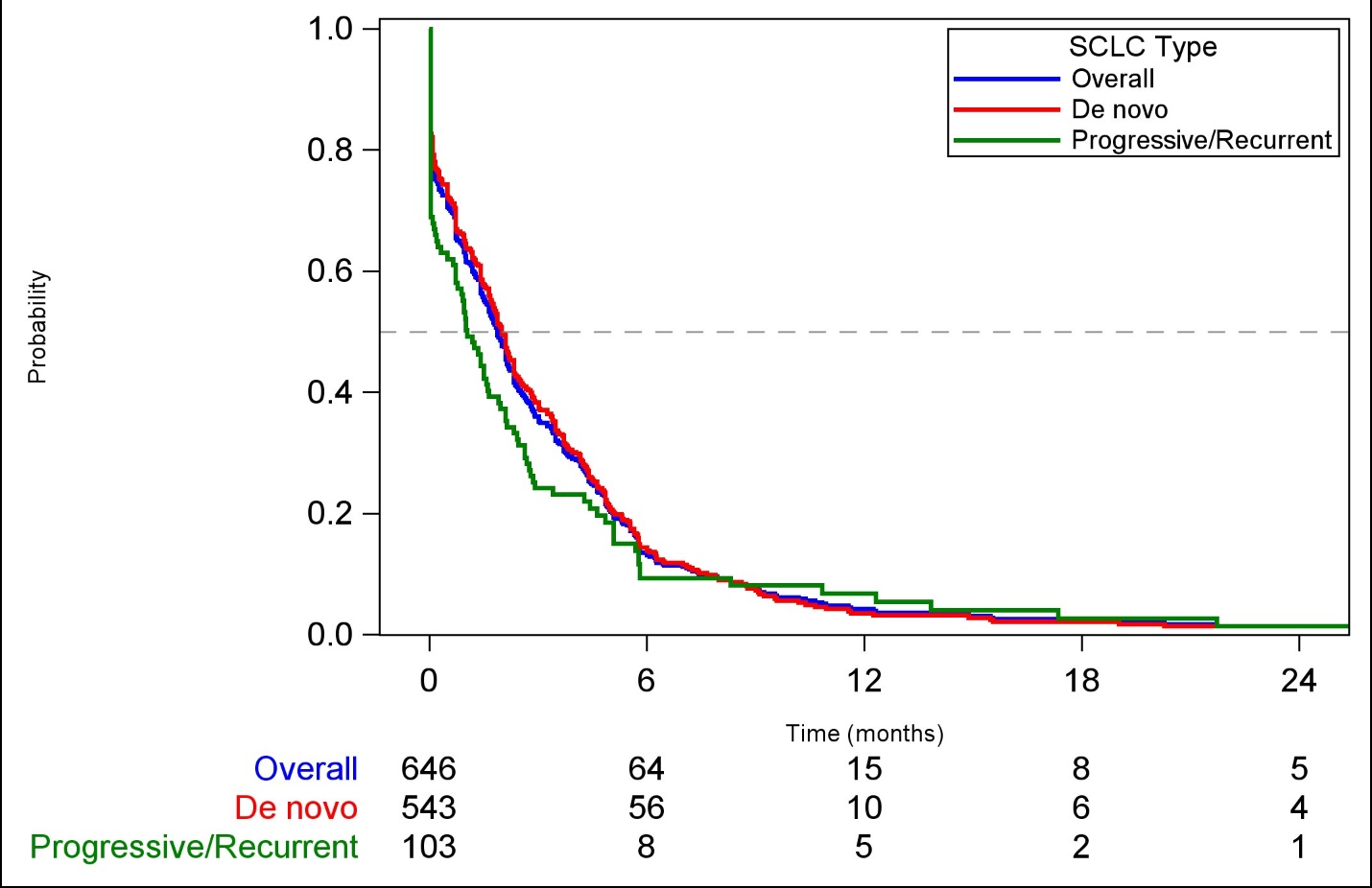


**Supplementary Figure 2.** Kaplan-Meier curves for real-world time to next treatment (rwTTNT), by ES-SCLC type, in A) first-line, B) second-line, and C) third-line therapy.

A)


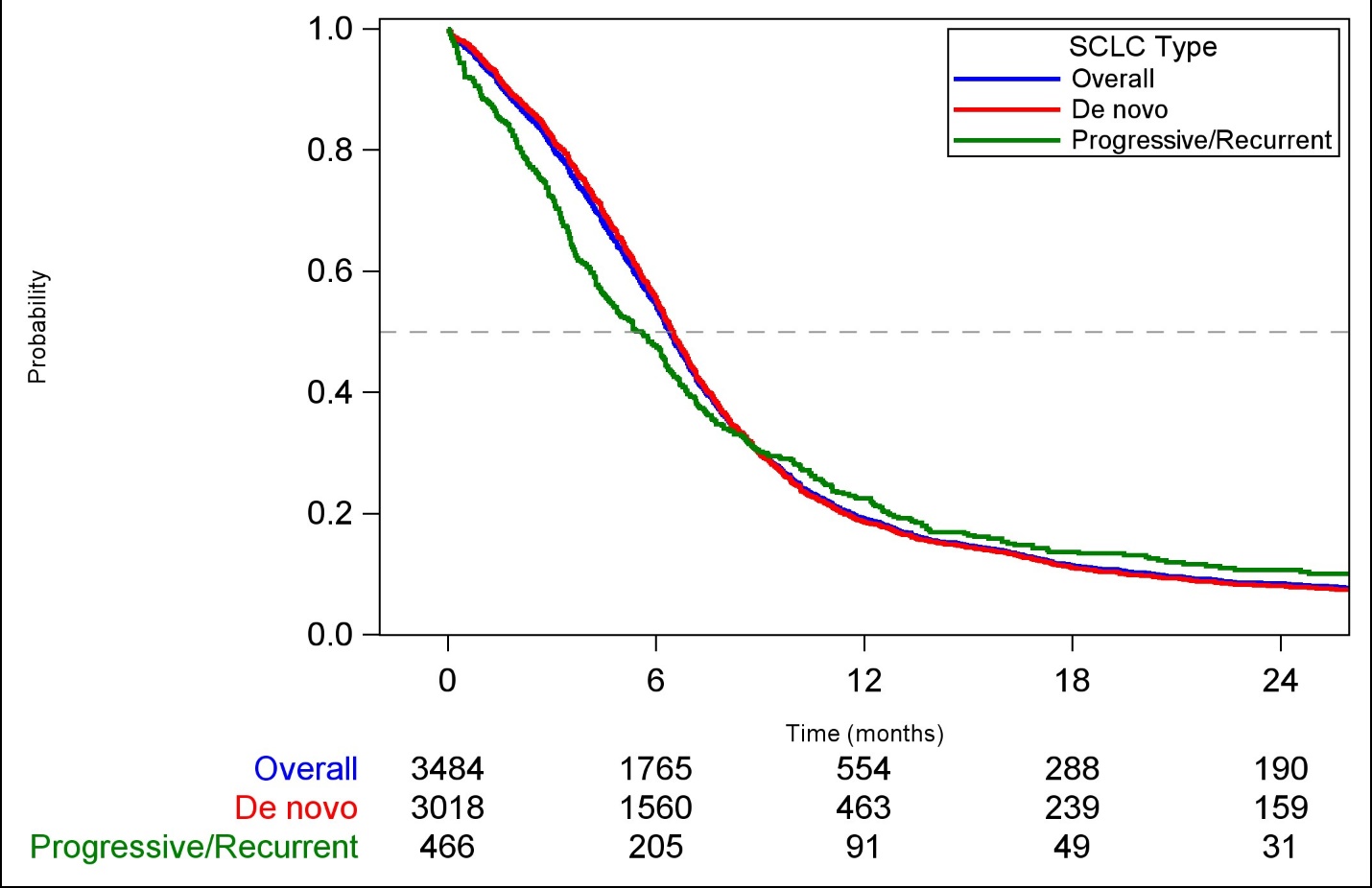


B)


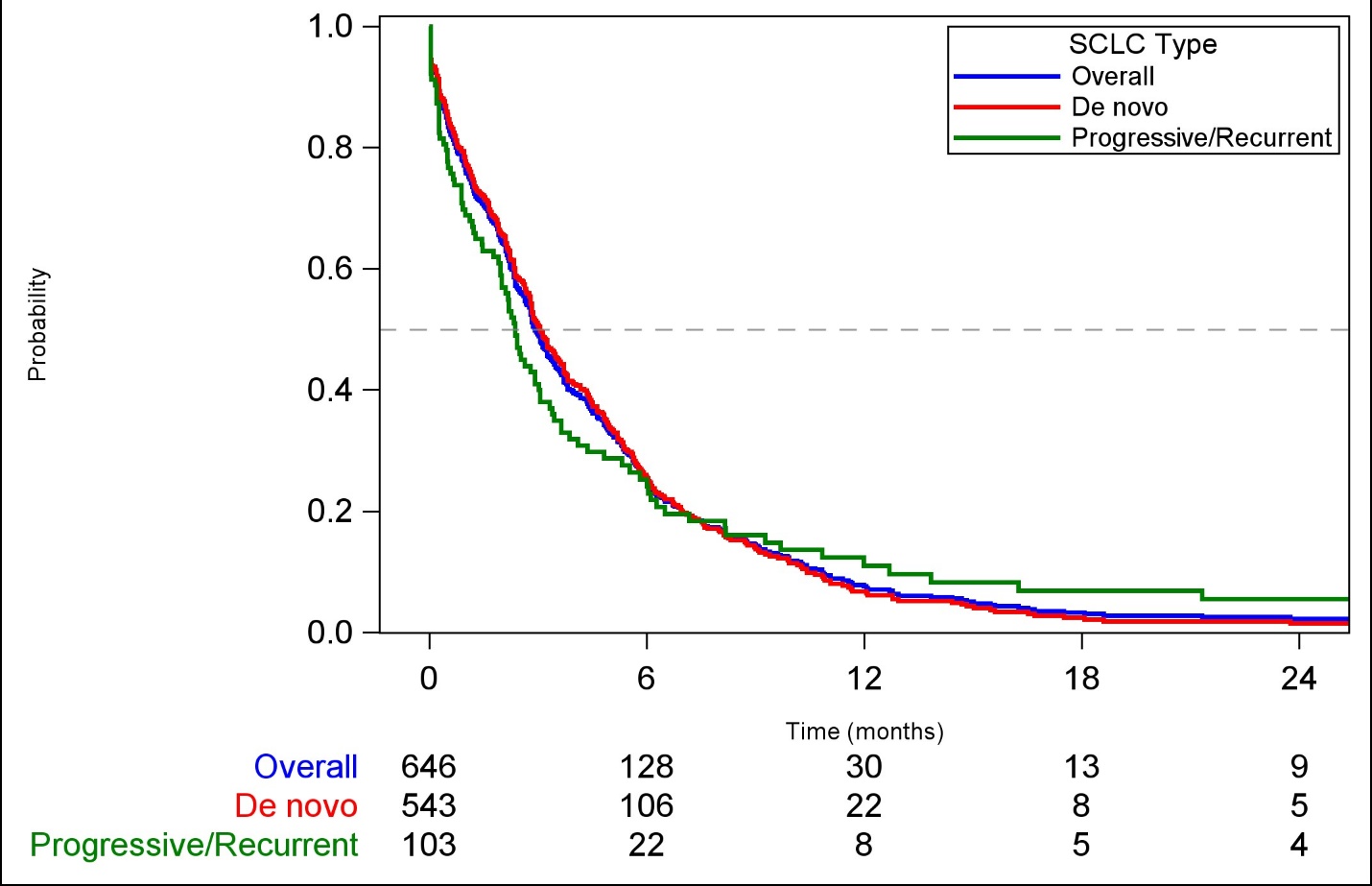


C)


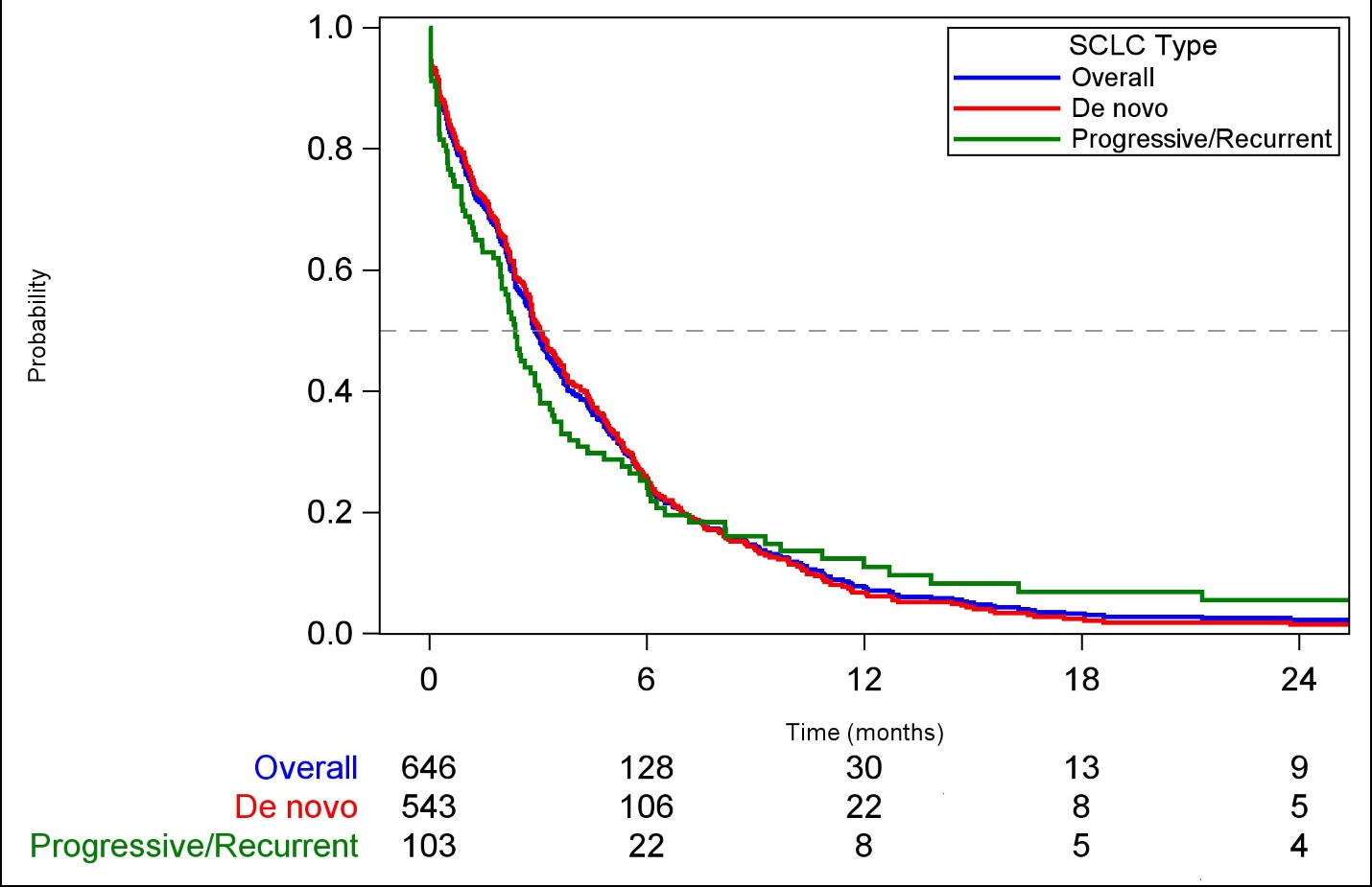


**Supplementary Figure 3.** Kaplan-Meier curves for real-world overall survival (rwOS), by ES-SCLC type, in A) first-line, B) second-line, and C) third-line therapy.

A)


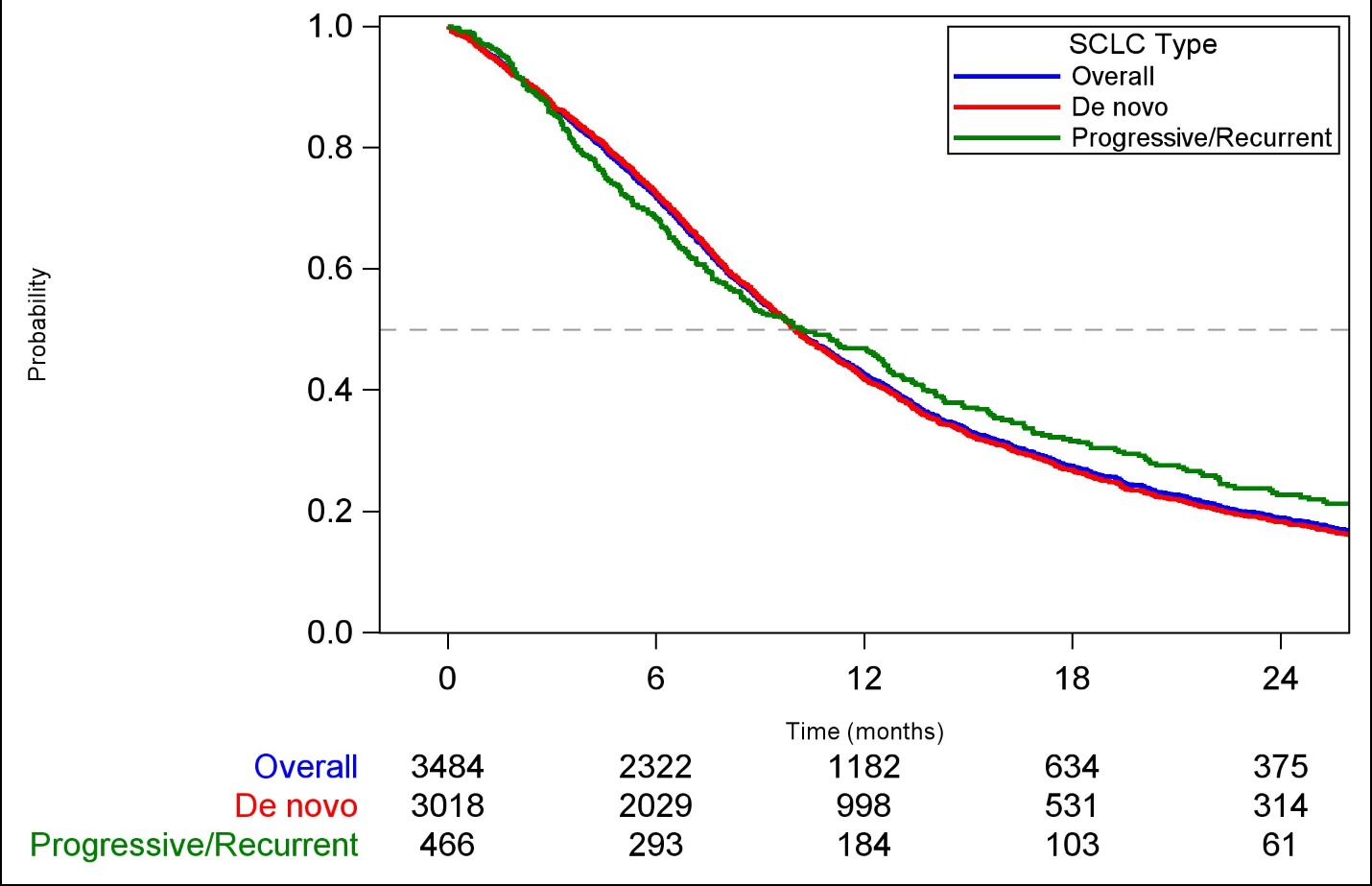


B)


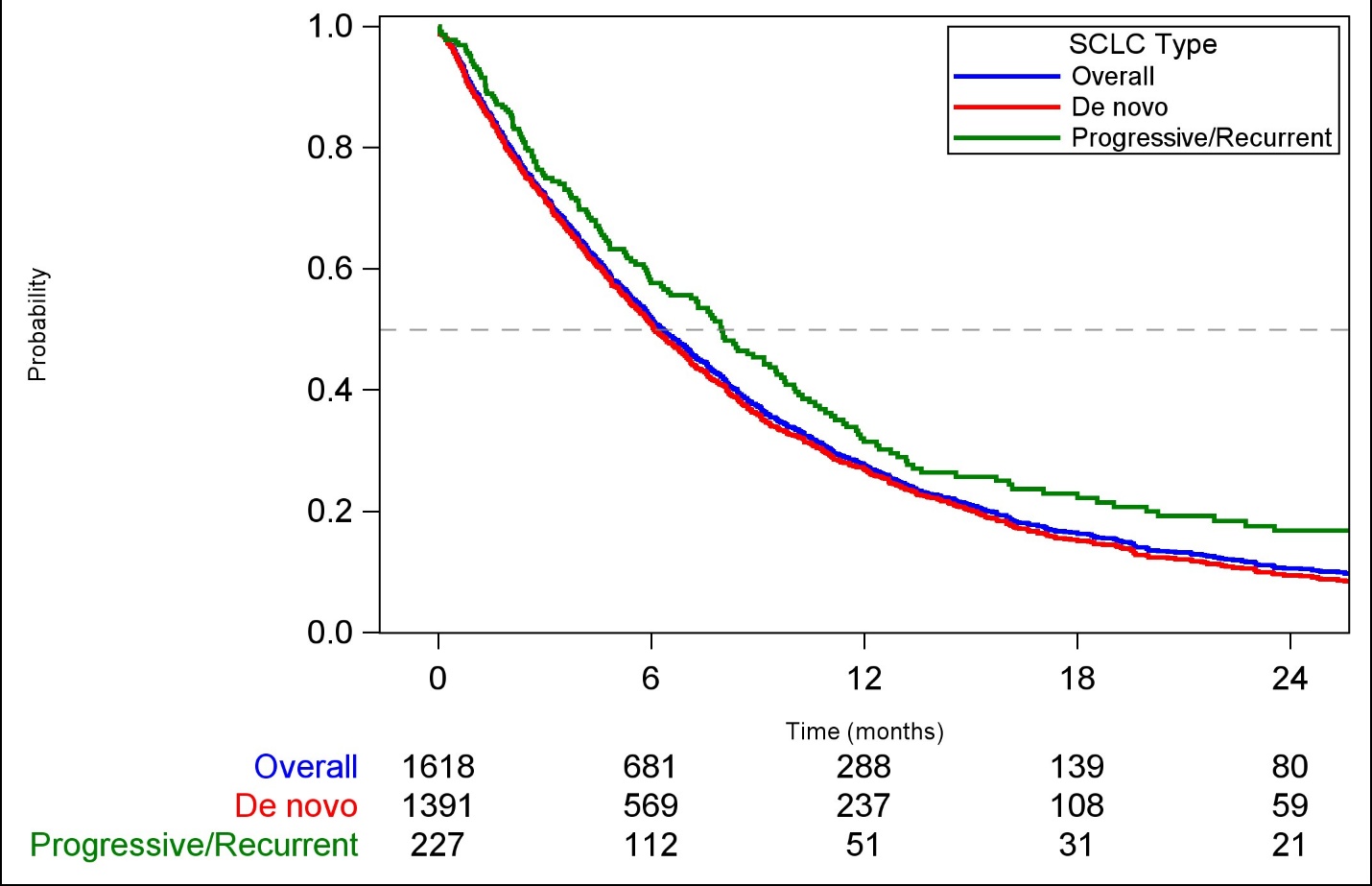


C)


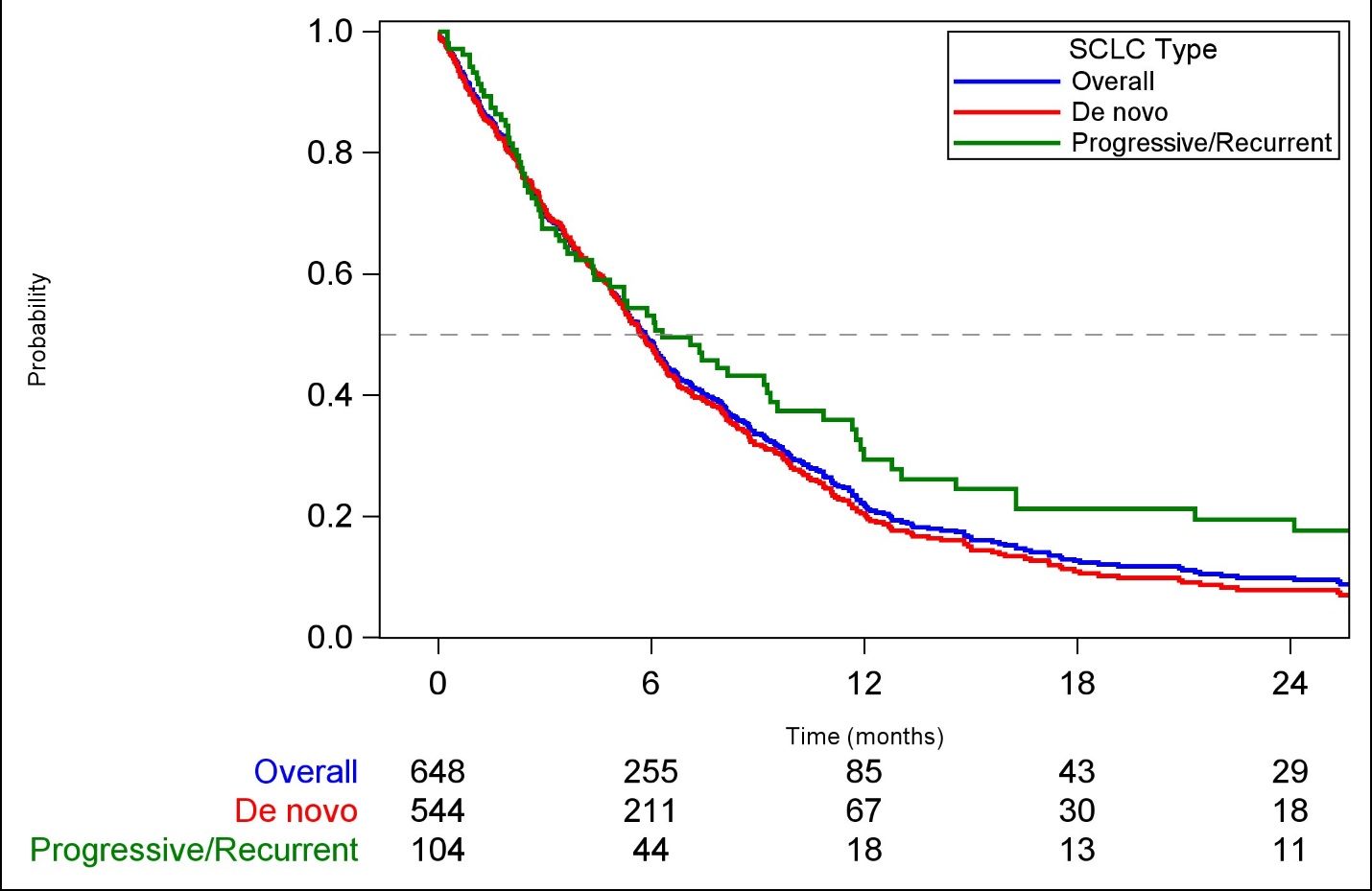


# Supplementary Tables

**Supplementary Table 1.** 1L to 3L treatment sequences for A) the overall ES-SCLC population, B) the de novo ES-SCLC population, C) the progressive/recurrent ES-SCLC population.

A)

| **Overall ES-SCLC Population (n=3484)** | | | | | | | | |
| --- | --- | --- | --- | --- | --- | --- | --- | --- |
| **1L Treatment Class** | **n** | **%** | **2L Treatment Class** | **n** | **%** | **3L Treatment Class** | **n** | **%** |
| ICI + Platinum-Based Chemotherapy | 2556 | 73.39 | No Further Treatment | 1483 | 58.00 | No Further Treatment | 1483 | 100.00 |
|  |  |  | Non-Platinum-Based Chemotherapy | 758 | 29.64 | No Further Treatment | 515 | 67.94 |
|  |  |  |  |  |  | Non-Platinum-Based Chemotherapy | 177 | 23.35 |
|  |  |  |  |  |  | ICI Monotherapy | 23 | 3.03 |
|  |  |  |  |  |  | Platinum-Based Chemotherapy | 22 | 2.90 |
|  |  |  |  |  |  | ICI + Platinum-Based Chemotherapy | 9 | 1.19 |
|  |  |  |  |  |  | ICI – ICI Combination Therapy | 5 | 0.66 |
|  |  |  |  |  |  | ICI + Other Chemotherapy | 3 | 0.40 |
|  |  |  |  |  |  | Targeted Therapy | 2 | 0.26 |
|  |  |  |  |  |  | Other | 2 | 0.26 |
|  |  |  | Platinum-Based Chemotherapy | 110 | 4.26 | No Further Treatment | 46 | 41.82 |
|  |  |  |  |  |  | Non-Platinum-Based Chemotherapy | 35 | 31.82 |
|  |  |  |  |  |  | ICI Monotherapy | 12 | 10.91 |
|  |  |  |  |  |  | Platinum-Based Chemotherapy | 8 | 7.27 |
|  |  |  |  |  |  | ICI + Platinum-Based Chemotherapy | 4 | 3.64 |
|  |  |  |  |  |  | ICI – ICI Combination Therapy | 3 | 2.73 |
|  |  |  |  |  |  | ICI + Other Chemotherapy | 1 | 0.91 |
|  |  |  |  |  |  | Other | 1 | 0.91 |
|  |  |  | ICI Monotherapy | 63 | 2.46 | No Further Treatment | 44 | 69.84 |
|  |  |  |  |  |  | Non-Platinum-Based Chemotherapy | 9 | 14.29 |
|  |  |  |  |  |  | Platinum-Based Chemotherapy | 4 | 6.35 |
|  |  |  |  |  |  | ICI + Platinum-Based Chemotherapy | 3 | 4.76 |
|  |  |  |  |  |  | ICI Monotherapy | 3 | 4.76 |
|  |  |  | ICI + Platinum-Based Chemotherapy | 59 | 2.31 | No Further Treatment | 26 | 44.07 |
|  |  |  |  |  |  | Non-Platinum-Based Chemotherapy | 16 | 27.12 |
|  |  |  |  |  |  | ICI + Other Chemotherapy | 5 | 8.47 |
|  |  |  |  |  |  | ICI + Platinum-Based Chemotherapy | 4 | 6.78 |
|  |  |  |  |  |  | Other | 4 | 6.78 |
|  |  |  |  |  |  | ICI Monotherapy | 2 | 3.39 |
|  |  |  |  |  |  | ICI – ICI Combination Therapy | 1 | 1.67 |
|  |  |  |  |  |  | Platinum-Based Chemotherapy | 1 | 1.67 |
|  |  |  | ICI + Other Chemotherapy | 39 | 1.53 | No Further Treatment | 29 | 74.36 |
|  |  |  |  |  |  | Non-Platinum-Based Chemotherapy | 8 | 20.51 |
|  |  |  |  |  |  | ICI + Platinum-Based Chemotherapy | 1 | 2.56 |
|  |  |  |  |  |  | ICI – ICI Combination Therapy | 1 | 2.56 |
|  |  |  | Other | 30 | 1.17 | ICI + Platinum-Based Chemotherapy | 16 | 53.33 |
|  |  |  |  |  |  | No Further Treatment | 8 | 26.67 |
|  |  |  |  |  |  | Non-Platinum-Based Chemotherapy | 3 | 10.00 |
|  |  |  |  |  |  | ICI Monotherapy | 2 | 6.67 |
|  |  |  |  |  |  | Platinum-Based Chemotherapy | 1 | 3.33 |
|  |  |  | ICI – ICI Combination Therapy | 15 | 0.59 | No Further Treatment | 12 | 80.00 |
|  |  |  |  |  |  | Non-Platinum-Based Chemotherapy | 2 | 13.33 |
|  |  |  |  |  |  | ICI Monotherapy | 1 | 6.67 |
| Platinum-Based Chemotherapy | 574 | 16.48 | No Further Treatment | 178 | 31.06 | No Further Treatment | 178 | 100.00 |
|  |  |  | ICI + Platinum-Based Chemotherapy | 153 | 26.70 | No Further Treatment | 77 | 50.33 |
|  |  |  |  |  |  | Non-Platinum-Based Chemotherapy | 50 | 32.68 |
|  |  |  |  |  |  | ICI Monotherapy | 11 | 7.19 |
|  |  |  |  |  |  | ICI + Platinum-Based Chemotherapy | 7 | 4.58 |
|  |  |  |  |  |  | Other | 4 | 2.61 |
|  |  |  |  |  |  | Platinum-Based Chemotherapy | 2 | 1.31 |
|  |  |  |  |  |  | ICI + Other Chemotherapy | 1 | 0.65 |
|  |  |  |  |  |  | ICI – ICI Combination Therapy | 1 | 0.65 |
|  |  |  | ICI Monotherapy | 127 | 22.16 | No Further Treatment | 70 | 55.12 |
|  |  |  |  |  |  | Non-Platinum-Based Chemotherapy | 27 | 21.26 |
|  |  |  |  |  |  | Platinum-Based Chemotherapy | 13 | 10.24 |
|  |  |  |  |  |  | ICI + Platinum-Based Chemotherapy | 11 | 8.66 |
|  |  |  |  |  |  | ICI + Other Chemotherapy | 2 | 1.57 |
|  |  |  |  |  |  | ICI Monotherapy | 2 | 1.57 |
|  |  |  |  |  |  | ICI – ICI Combination Therapy | 1 | 0.79 |
|  |  |  |  |  |  | Other | 1 | 0.79 |
|  |  |  | Non-Platinum-Based Chemotherapy | 59 | 10.308 | No Further Treatment | 35 | 59.32 |
|  |  |  |  |  |  | ICI Monotherapy | 10 | 16.95 |
|  |  |  |  |  |  | Non-Platinum-Based Chemotherapy | 10 | 16.95 |
|  |  |  |  |  |  | ICI + Platinum-Based Chemotherapy | 2 | 3.39 |
|  |  |  |  |  |  | Platinum-Based Chemotherapy | 2 | 3.39 |
|  |  |  | Platinum-Based Chemotherapy | 25 | 4.36 | No Further Treatment | 8 | 32.00 |
|  |  |  |  |  |  | ICI + Platinum-Based Chemotherapy | 5 | 20.00 |
|  |  |  |  |  |  | ICI Monotherapy | 4 | 16.00 |
|  |  |  |  |  |  | Non-Platinum-Based Chemotherapy | 4 | 16.00 |
|  |  |  |  |  |  | Platinum-Based Chemotherapy | 4 | 16.00 |
|  |  |  | ICI – ICI Combination Therapy | 18 | 3.14 | No Further Treatment | 13 | 72.22 |
|  |  |  |  |  |  | Non-Platinum-Based Chemotherapy | 3 | 16.67 |
|  |  |  |  |  |  | ICI Monotherapy | 2 | 11.11 |
|  |  |  | ICI + Other Chemotherapy | 7 | 1.22 | No Further Treatment | 3 | 42.86 |
|  |  |  |  |  |  | ICI + Platinum-Based Chemotherapy | 2 | 28.57 |
|  |  |  |  |  |  | Non-Platinum-Based Chemotherapy | 1 | 14.29 |
|  |  |  |  |  |  | Platinum-Based Chemotherapy | 1 | 14.29 |
|  |  |  | Other | 6 | 1.05 | No Further Treatment | 2 | 33.33 |
|  |  |  |  |  |  | ICI + Platinum-Based Chemotherapy | 1 | 16.67 |
|  |  |  |  |  |  | ICI – ICI Combination Therapy | 1 | 16.67 |
|  |  |  |  |  |  | Other | 1 | 16.67 |
|  |  |  |  |  |  | Platinum-Based Chemotherapy | 1 | 16.67 |
| ICI Monotherapy | 186 | 5.34 | No Further Treatment | 111 | 59.36 | No Further Treatment | 111 | 100.00 |
|  |  |  | Non-Platinum-Based Chemotherapy | 38 | 20.32 | No Further Treatment | 22 | 57.89 |
|  |  |  |  |  |  | Non-Platinum-Based Chemotherapy | 11 | 28.95 |
|  |  |  |  |  |  | ICI Monotherapy | 3 | 7.89 |
|  |  |  |  |  |  | ICI + Platinum-Based Chemotherapy | 1 | 2.63 |
|  |  |  |  |  |  | ICI – ICI Combination Therapy | 1 | 2.63 |
|  |  |  | Platinum-Based Chemotherapy | 18 | 9.63 | No Further Treatment | 6 | 33.33 |
|  |  |  |  |  |  | ICI Monotherapy | 4 | 22.22 |
|  |  |  |  |  |  | Non-Platinum-Based Chemotherapy | 4 | 22.22 |
|  |  |  |  |  |  | Platinum-Based Chemotherapy | 2 | 11.11 |
|  |  |  |  |  |  | ICI + Platinum-Based Chemotherapy | 1 | 5.56 |
|  |  |  |  |  |  | ICI – ICI Combination Therapy | 1 | 5.56 |
|  |  |  | ICI + Platinum-Based Chemotherapy | 6 | 3.23 | No Further Treatment | 3 | 50.00 |
|  |  |  |  |  |  | Non-Platinum-Based Chemotherapy | 2 | 33.33 |
|  |  |  |  |  |  | Platinum-Based Chemotherapy | 1 | 16.67 |
|  |  |  | ICI Monotherapy | 6 | 3.23 | No Further Treatment | 4 | 66.67 |
|  |  |  |  |  |  | ICI + Other Chemotherapy | 1 | 16.67 |
|  |  |  |  |  |  | Non-Platinum-Based Chemotherapy | 1 | 16.67 |
|  |  |  | Other | 5 | 2.67 | ICI Monotherapy | 2 | 40.00 |
|  |  |  |  |  |  | No Further Treatment | 2 | 40.00 |
|  |  |  |  |  |  | Non-Platinum-Based Chemotherapy | 1 | 20.00 |
|  |  |  | ICI + Other Chemotherapy | 3 | 1.61 | Non-Platinum-Based Chemotherapy | 2 | 66.67 |
|  |  |  |  |  |  | ICI + Other Chemotherapy | 1 | 33.33 |
| Non-Platinum-Based Chemotherapy | 108 | 3.10 | No Further Treatment | 63 | 58.33 | No Further Treatment | 63 | 100.00 |
|  |  |  | Non-Platinum-Based Chemotherapy | 20 | 18.52 | No Further Treatment | 14 | 70.00 |
|  |  |  |  |  |  | ICI Monotherapy | 4 | 20.00 |
|  |  |  |  |  |  | Non-Platinum-Based Chemotherapy | 1 | 5.00 |
|  |  |  |  |  |  | Other | 1 | 5.00 |
|  |  |  | ICI Monotherapy | 16 | 14.81 | No Further Treatment | 10 | 62.50 |
|  |  |  |  |  |  | Non-Platinum-Based Chemotherapy | 6 | 37.50 |
|  |  |  | Platinum-Based Chemotherapy | 4 | 3.70 | No Further Treatment | 3 | 75.00 |
|  |  |  |  |  |  | Non-Platinum-Based Chemotherapy | 1 | 25.00 |
|  |  |  | ICI + Platinum-Based Chemotherapy | 2 | 1.85 | ICI – ICI Combination Therapy | 1 | 50.00 |
|  |  |  |  |  |  | No Further Treatment | 1 | 50.00 |
|  |  |  | ICI + Other Chemotherapy | 1 | 0.93 | No Further Treatment | 1 | 100.00 |
|  |  |  | ICI – ICI Combination Therapy | 1 | 0.93 | ICI + Platinum-Based Chemotherapy | 1 | 100.00 |
|  |  |  | Other | 1 | 0.93 | No Further Treatment | 1 | 100.00 |
| ICI – ICI Combination Therapy | 35 | 1.00 | No Further Treatment | 23 | 65.71 | No Further Treatment | 23 | 100.00 |
|  |  |  | Non-Platinum-Based Chemotherapy | 8 | 22.86 | Non-Platinum-Based Chemotherapy | 5 | 62.50 |
|  |  |  |  |  |  | No Further Treatment | 2 | 25.00 |
|  |  |  |  |  |  | Other | 1 | 12.50 |
|  |  |  | ICI Monotherapy | 3 | 8.57 | No Further Treatment | 3 | 100.00 |
|  |  |  | Platinum-Based Chemotherapy | 1 | 2.86 | No Further Treatment | 1 | 100.00 |
| Other | 16 | 0.46 | No Further Treatment | 5 | 31.25 | No Further Treatment | 5 | 100.00 |
|  |  |  | Platinum-Based Chemotherapy | 4 | 25.00 | ICI Monotherapy | 2 | 50.00 |
|  |  |  |  |  |  | ICI – ICI Combination Therapy | 1 | 25.00 |
|  |  |  |  |  |  | No Further Treatment | 1 | 25.00 |
|  |  |  | ICI Monotherapy | 3 | 18.75 | No Further Treatment | 1 | 33.33 |
|  |  |  |  |  |  | Non-Platinum-Based Chemotherapy | 1 | 33.33 |
|  |  |  |  |  |  | Platinum-Based Chemotherapy | 1 | 33.33 |
|  |  |  | Non-Platinum-Based Chemotherapy | 2 | 12.50 | No Further Treatment | 2 | 100.00 |
|  |  |  | ICI + Platinum-Based Chemotherapy | 1 | 6.25 | No Further Treatment | 1 | 100.00 |
|  |  |  | ICI – ICI Combination Therapy | 1 | 6.25 | No Further Treatment | 1 | 100.00 |
| ICI + Other Chemotherapy | 8 | 0.23 | No Further Treatment | 5 | 62.50 | No Further Treatment | 5 | 100.00 |
|  |  |  | ICI Monotherapy | 2 | 25.00 | No Further Treatment | 2 | 100.00 |
|  |  |  | Non-Platinum-Based Chemotherapy | 1 | 12.50 | No Further Treatment | 1 | 100.00 |

**Abbreviations:** 1L, first-line; 2L, second-line; 3L, third-line; ES-SCLC, extensive-stage small cell lung cancer; ICI, immune checkpoint inhibitor.

B)

| **De Novo ES-SCLC Population (n=3018)** | | | | | | | | |
| --- | --- | --- | --- | --- | --- | --- | --- | --- |
| **1L Treatment Class** | **n** | **%** | **2L Treatment Class** | **n** | **%** | **3L Treatment Class** | **n** | **%** |
| ICI + Platinum-Based Chemotherapy | 2448 | 81.11 | No Further Treatment | 1431 | 58.46 | No Further Treatment | 1431 | 100.00 |
|  |  |  | Non-Platinum-Based Chemotherapy | 720 | 29.41 | No Further Treatment | 489 | 67.92 |
|  |  |  |  |  |  | Non-Platinum-Based Chemotherapy | 168 | 23.33 |
|  |  |  |  |  |  | ICI Monotherapy | 22 | 3.06 |
|  |  |  |  |  |  | Platinum-Based Chemotherapy | 21 | 2.92 |
|  |  |  |  |  |  | ICI + Platinum-Based Chemotherapy | 9 | 1.25 |
|  |  |  |  |  |  | ICI – ICI Combination Therapy | 4 | 0.56 |
|  |  |  |  |  |  | ICI + Other Chemotherapy | 3 | 0.42 |
|  |  |  |  |  |  | Targeted Therapy | 2 | 0.28 |
|  |  |  |  |  |  | Other | 2 | 0.28 |
|  |  |  | Platinum-Based Chemotherapy | 98 | 4.00 | No Further Treatment | 41 | 41.84 |
|  |  |  |  |  |  | Non-Platinum-Based Chemotherapy | 33 | 33.67 |
|  |  |  |  |  |  | ICI Monotherapy | 10 | 10.20 |
|  |  |  |  |  |  | Platinum-Based Chemotherapy | 8 | 8.16 |
|  |  |  |  |  |  | ICI – ICI Combination Therapy | 3 | 3.06 |
|  |  |  |  |  |  | ICI + Platinum-Based Chemotherapy | 2 | 2.04 |
|  |  |  |  |  |  | ICI + Other Chemotherapy | 1 | 1.02 |
|  |  |  | ICI Monotherapy | 62 | 2.53 | No Further Treatment | 43 | 69.35 |
|  |  |  |  |  |  | Non-Platinum-Based Chemotherapy | 9 | 14.52 |
|  |  |  |  |  |  | Platinum-Based Chemotherapy | 4 | 6.45 |
|  |  |  |  |  |  | ICI + Platinum-Based Chemotherapy | 3 | 4.84 |
|  |  |  |  |  |  | ICI Monotherapy | 3 | 4.84 |
|  |  |  | ICI + Platinum-Based Chemotherapy | 55 | 2.25 | No Further Treatment | 24 | 43.64 |
|  |  |  |  |  |  | Non-Platinum-Based Chemotherapy | 16 | 29.09 |
|  |  |  |  |  |  | ICI + Other Chemotherapy | 4 | 7.27 |
|  |  |  |  |  |  | ICI + Platinum-Based Chemotherapy | 4 | 7.27 |
|  |  |  |  |  |  | Other | 4 | 7.27 |
|  |  |  |  |  |  | ICI – ICI Combination Therapy | 1 | 1.82 |
|  |  |  |  |  |  | ICI Monotherapy | 1 | 1.82 |
|  |  |  |  |  |  | Platinum-Based Chemotherapy | 1 | 1.82 |
|  |  |  | ICI + Other Chemotherapy | 39 | 1.59 | No Further Treatment | 29 | 74.36 |
|  |  |  |  |  |  | Non-Platinum-Based Chemotherapy | 8 | 20.51 |
|  |  |  |  |  |  | ICI + Platinum-Based Chemotherapy | 1 | 2.56 |
|  |  |  |  |  |  | ICI – ICI Combination Therapy | 1 | 2.56 |
|  |  |  | Other | 29 | 1.18 | ICI + Platinum-Based Chemotherapy | 15 | 51.72 |
|  |  |  |  |  |  | No Further Treatment | 8 | 27.59 |
|  |  |  |  |  |  | Non-Platinum-Based Chemotherapy | 3 | 10.34 |
|  |  |  |  |  |  | ICI Monotherapy | 2 | 6.90 |
|  |  |  |  |  |  | Platinum-Based Chemotherapy | 1 | 3.45 |
|  |  |  | ICI – ICI Combination Therapy | 14 | 0.57 | No Further Treatment | 12 | 85.71 |
|  |  |  |  |  |  | Non-Platinum-Based Chemotherapy | 2 | 14.29 |
| Platinum-Based Chemotherapy | 485 | 16.07 | No Further Treatment | 151 | 31.13 | No Further Treatment | 151 | 100.00 |
|  |  |  | ICI + Platinum-Based Chemotherapy | 132 | 27.22 | No Further Treatment | 65 | 49.24 |
|  |  |  |  |  |  | Non-Platinum-Based Chemotherapy | 42 | 31.82 |
|  |  |  |  |  |  | ICI Monotherapy | 11 | 8.33 |
|  |  |  |  |  |  | ICI + Platinum-Based Chemotherapy | 6 | 4.55 |
|  |  |  |  |  |  | Other | 4 | 3.03 |
|  |  |  |  |  |  | Platinum-Based Chemotherapy | 2 | 1.52 |
|  |  |  |  |  |  | ICI + Other Chemotherapy | 1 | 0.76 |
|  |  |  |  |  |  | ICI – ICI Combination Therapy | 1 | 0.76 |
|  |  |  | ICI Monotherapy | 106 | 21.86 | No Further Treatment | 62 | 58.49 |
|  |  |  |  |  |  | Non-Platinum-Based Chemotherapy | 22 | 20.75 |
|  |  |  |  |  |  | ICI + Platinum-Based Chemotherapy | 10 | 9.43 |
|  |  |  |  |  |  | Platinum-Based Chemotherapy | 8 | 7.55 |
|  |  |  |  |  |  | ICI Monotherapy | 2 | 1.89 |
|  |  |  |  |  |  | ICI + Other Chemotherapy | 1 | 0.94 |
|  |  |  |  |  |  | ICI – ICI Combination Therapy | 1 | 0.94 |
|  |  |  | Non-Platinum-Based Chemotherapy | 48 | 9.90 | No Further Treatment | 31 | 64.58 |
|  |  |  |  |  |  | Non-Platinum-Based Chemotherapy | 8 | 16.67 |
|  |  |  |  |  |  | ICI Monotherapy | 5 | 10.42 |
|  |  |  |  |  |  | ICI + Platinum-Based Chemotherapy | 2 | 4.17 |
|  |  |  |  |  |  | Platinum-Based Chemotherapy | 2 | 4.17 |
|  |  |  | ICI – ICI Combination Therapy | 18 | 3.71 | No Further Treatment | 13 | 72.22 |
|  |  |  |  |  |  | Non-Platinum-Based Chemotherapy | 3 | 16.67 |
|  |  |  |  |  |  | ICI Monotherapy | 2 | 11.11 |
|  |  |  | Platinum-Based Chemotherapy | 18 | 3.71 | No Further Treatment | 7 | 38.89 |
|  |  |  |  |  |  | ICI Monotherapy | 3 | 16.67 |
|  |  |  |  |  |  | Non-Platinum-Based Chemotherapy | 3 | 16.67 |
|  |  |  |  |  |  | Platinum-Based Chemotherapy | 3 | 16.67 |
|  |  |  |  |  |  | ICI + Platinum-Based Chemotherapy | 2 | 11.11 |
|  |  |  | ICI + Other Chemotherapy | 7 | 1.44 | No Further Treatment | 3 | 42.86 |
|  |  |  |  |  |  | ICI + Platinum-Based Chemotherapy | 2 | 28.57 |
|  |  |  |  |  |  | Non-Platinum-Based Chemotherapy | 1 | 14.29 |
|  |  |  |  |  |  | Platinum-Based Chemotherapy | 1 | 14.29 |
|  |  |  | Other | 5 | 1.03 | ICI + Platinum-Based Chemotherapy | 1 | 20.00 |
|  |  |  |  |  |  | ICI – ICI Combination Therapy | 1 | 20.00 |
|  |  |  |  |  |  | No Further Treatment | 1 | 20.00 |
|  |  |  |  |  |  | Other | 1 | 20.00 |
|  |  |  |  |  |  | Platinum-Based Chemotherapy | 1 | 20.00 |
| ICI Monotherapy | 49 | 1.62 | No Further Treatment | 25 | 50.00 | No Further Treatment | 25 | 100.00 |
|  |  |  | Non-Platinum-Based Chemotherapy | 13 | 26.00 | No Further Treatment | 8 | 61.54 |
|  |  |  |  |  |  | Non-Platinum-Based Chemotherapy | 4 | 30.77 |
|  |  |  |  |  |  | ICI Monotherapy | 1 | 7.69 |
|  |  |  | Platinum-Based Chemotherapy | 6 | 12.00 | Non-Platinum-Based Chemotherapy | 2 | 33.33 |
|  |  |  |  |  |  | ICI – ICI Combination Therapy | 1 | 16.67 |
|  |  |  |  |  |  | ICI Monotherapy | 1 | 16.67 |
|  |  |  |  |  |  | No Further Treatment | 1 | 16.67 |
|  |  |  |  |  |  | Platinum-Based Chemotherapy | 1 | 16.67 |
|  |  |  | ICI + Platinum-Based Chemotherapy | 3 | 6.00 | Non-Platinum-Based Chemotherapy | 2 | 66.67 |
|  |  |  |  |  |  | No Further Treatment | 1 | 33.33 |
|  |  |  | Other | 2 | 4.00 | ICI Monotherapy | 1 | 50.00 |
|  |  |  |  |  |  | Non-Platinum-Based Chemotherapy | 1 | 50.00 |
|  |  |  | ICI + Other Chemotherapy | 1 | 2.00 | Non-Platinum-Based Chemotherapy | 1 | 100.00 |
| Non-Platinum-Based Chemotherapy | 24 | 0.80 | No Further Treatment | 14 | 58.33 | No Further Treatment | 14 | 100.00 |
|  |  |  | Non-Platinum-Based Chemotherapy | 5 | 20.83 | No Further Treatment | 4 | 80.00 |
|  |  |  |  |  |  | Non-Platinum-Based Chemotherapy | 1 | 20.00 |
|  |  |  | ICI Monotherapy | 2 | 8.33 | No Further Treatment | 2 | 100.00 |
|  |  |  | ICI + Platinum-Based Chemotherapy | 1 | 4.17 | ICI – ICI Combination Therapy | 1 | 100.00 |
|  |  |  | Other | 1 | 4.17 | No Further Treatment | 1 | 100.00 |
|  |  |  | Platinum-Based Chemotherapy | 1 | 4.17 | Non-Platinum-Based Chemotherapy | 1 | 100.00 |
| ICI – ICI Combination Therapy | 5 | 0.17 | No Further Treatment | 4 | 80.00 | No Further Treatment | 4 | 100.00 |
|  |  |  | ICI Monotherapy | 1 | 20.00 | No Further Treatment | 1 | 100.00 |
| Other | 5 | 0.17 | No Further Treatment | 2 | 40.00 | No Further Treatment | 2 | 100.00 |
|  |  |  | Platinum-Based Chemotherapy | 2 | 40.00 | ICI Monotherapy | 1 | 50.00 |
|  |  |  |  |  |  | ICI – ICI Combination Therapy | 1 | 50.00 |
|  |  |  | Non-Platinum-Based Chemotherapy | 1 | 20.00 | No Further Treatment | 1 | 100.00 |
| ICI + Other Chemotherapy | 1 | 0.03 | No Further Treatment | 1 | 100.00 | No Further Treatment | 1 | 100.00 |

**Abbreviations:** 1L, first-line; 2L, second-line; 3L, third-line; ES-SCLC, extensive-stage small cell lung cancer; ICI, immune checkpoint inhibitor.

C)

| **Progressive/Recurrent ES-SCLC Population (n=466)** | | | | | | | | |
| --- | --- | --- | --- | --- | --- | --- | --- | --- |
| **1L Treatment Class** | **n** | **%** | **2L Treatment Class** | **n** | **%** | **3L Treatment Class** | **n** | **%** |
| ICI Monotherapy | 137 | 29.40 | No Further Treatment | 86 | 62.77 | No Further Treatment | 86 | 100.00 |
|  |  |  | Non-Platinum-Based Chemotherapy | 25 | 18.25 | No Further Treatment | 14 | 56.00 |
|  |  |  |  |  |  | Non-Platinum-Based Chemotherapy | 7 | 28.00 |
|  |  |  |  |  |  | ICI Monotherapy | 2 | 8.00 |
|  |  |  |  |  |  | ICI + Platinum-Based Chemotherapy | 1 | 4.00 |
|  |  |  |  |  |  | ICI – ICI Combination Therapy | 1 | 4.00 |
|  |  |  | Platinum-Based Chemotherapy | 12 | 8.76 | No Further Treatment | 5 | 41.67 |
|  |  |  |  |  |  | ICI Monotherapy | 3 | 25.00 |
|  |  |  |  |  |  | Non-Platinum-Based Chemotherapy | 2 | 16.67 |
|  |  |  |  |  |  | ICI + Platinum-Based Chemotherapy | 1 | 8.33 |
|  |  |  |  |  |  | Platinum-Based Chemotherapy | 1 | 8.33 |
|  |  |  | ICI Monotherapy | 6 | 4.38 | No Further Treatment | 4 | 66.67 |
|  |  |  |  |  |  | ICI + Other Chemotherapy | 1 | 16.67 |
|  |  |  |  |  |  | Non-Platinum-Based Chemotherapy | 1 | 16.67 |
|  |  |  | ICI + Platinum-Based Chemotherapy | 3 | 2.19 | No Further Treatment | 2 | 66.67 |
|  |  |  |  |  |  | Platinum-Based Chemotherapy | 1 | 33.33 |
|  |  |  | Other | 3 | 2.19 | No Further Treatment | 2 | 66.67 |
|  |  |  |  |  |  | ICI Monotherapy | 1 | 33.33 |
|  |  |  | ICI + Other Chemotherapy | 2 | 1.46 | ICI + Other Chemotherapy | 1 | 50.00 |
|  |  |  |  |  |  | Non-Platinum-Based Chemotherapy | 1 | 50.00 |
| ICI + Platinum-Based Chemotherapy | 109 | 23.39 | No Further Treatment | 52 | 47.71 | No Further Treatment | 52 | 100.00 |
|  |  |  | Non-Platinum-Based Chemotherapy | 38 | 34.86 | No Further Treatment | 26 | 68.42 |
|  |  |  |  |  |  | Non-Platinum-Based Chemotherapy | 9 | 23.68 |
|  |  |  |  |  |  | ICI – ICI Combination Therapy | 1 | 2.63 |
|  |  |  |  |  |  | ICI Monotherapy | 1 | 2.63 |
|  |  |  |  |  |  | Platinum-Based Chemotherapy | 1 | 2.63 |
|  |  |  | Platinum-Based Chemotherapy | 12 | 11.01 | No Further Treatment | 5 | 41.67 |
|  |  |  |  |  |  | ICI Monotherapy | 2 | 16.67 |
|  |  |  |  |  |  | Non-Platinum-Based Chemotherapy | 2 | 16.67 |
|  |  |  |  |  |  | ICI + Platinum-Based Chemotherapy | 2 | 16.67 |
|  |  |  |  |  |  | Other | 1 | 8.33 |
|  |  |  | ICI + Platinum-Based Chemotherapy | 4 | 3.67 | No Further Treatment | 2 | 50.00 |
|  |  |  |  |  |  | ICI + Other Chemotherapy | 1 | 25.00 |
|  |  |  |  |  |  | ICI Monotherapy | 1 | 25.00 |
|  |  |  | ICI – ICI Combination Therapy | 1 | 0.92 | ICI Monotherapy | 1 | 100.00 |
|  |  |  | ICI Monotherapy | 1 | 0.92 | No Further Treatment | 1 | 100.00 |
|  |  |  | Other | 1 | 0.92 | ICI + Platinum-Based Chemotherapy | 1 | 100.00 |
| Platinum-Based Chemotherapy | 88 | 18.88 | No Further Treatment | 27 | 30.34 | No Further Treatment | 27 | 100.00 |
|  |  |  | ICI + Platinum-Based Chemotherapy | 22 | 24.72 | No Further Treatment | 12 | 57.14 |
|  |  |  |  |  |  | Non-Platinum-Based Chemotherapy | 8 | 38.10 |
|  |  |  |  |  |  | ICI + Platinum-Based Chemotherapy | 1 | 4.76 |
|  |  |  | ICI Monotherapy | 21 | 23.86 | No Further Treatment | 8 | 38.10 |
|  |  |  |  |  |  | Non-Platinum-Based Chemotherapy | 5 | 23.81 |
|  |  |  |  |  |  | Platinum-Based Chemotherapy | 5 | 23.81 |
|  |  |  |  |  |  | ICI + Other Chemotherapy | 1 | 4.76 |
|  |  |  |  |  |  | ICI + Platinum-Based Chemotherapy | 1 | 4.76 |
|  |  |  |  |  |  | Other | 1 | 4.76 |
|  |  |  | Non-Platinum-Based Chemotherapy | 11 | 12.50 | ICI Monotherapy | 5 | 45.45 |
|  |  |  |  |  |  | No Further Treatment | 4 | 36.36 |
|  |  |  |  |  |  | Non-Platinum-Based Chemotherapy | 2 | 18.18 |
|  |  |  | Platinum-Based Chemotherapy | 7 | 7.95 | ICI + Platinum-Based Chemotherapy | 3 | 42.86 |
|  |  |  |  |  |  | ICI Monotherapy | 1 | 14.29 |
|  |  |  |  |  |  | No Further Treatment | 1 | 14.29 |
|  |  |  |  |  |  | Non-Platinum-Based Chemotherapy | 1 | 14.29 |
|  |  |  |  |  |  | Platinum-Based Chemotherapy | 1 | 14.29 |
|  |  |  | Other | 1 | 1.14 | No Further Treatment | 1 | 100.00 |
| Non-Platinum-Based Chemotherapy | 84 | 18.03 | No Further Treatment | 49 | 58.33 | No Further Treatment | 49 | 100.00 |
|  |  |  | Non-Platinum-Based Chemotherapy | 15 | 17.86 | No Further Treatment | 10 | 66.67 |
|  |  |  |  |  |  | ICI Monotherapy | 4 | 26.67 |
|  |  |  |  |  |  | Other | 1 | 6.67 |
|  |  |  | ICI Monotherapy | 14 | 16.67 | No Further Treatment | 8 | 57.14 |
|  |  |  |  |  |  | Non-Platinum-Based Chemotherapy | 6 | 42.86 |
|  |  |  | Platinum-Based Chemotherapy | 3 | 3.57 | No Further Treatment | 3 | 100.00 |
|  |  |  | ICI + Other Chemotherapy | 1 | 1.19 | No Further Treatment | 1 | 100.00 |
|  |  |  | ICI + Platinum-Based Chemotherapy | 1 | 1.19 | No Further Treatment | 1 | 100.00 |
|  |  |  | ICI – ICI Combination Therapy | 1 | 1.19 | ICI + Platinum-Based Chemotherapy | 1 | 100.00 |
| ICI – ICI Combination Therapy | 30 | 6.44 | No Further Treatment | 19 | 63.33 | No Further Treatment | 19 | 100.00 |
|  |  |  | Non-Platinum-Based Chemotherapy | 8 | 26.67 | Non-Platinum-Based Chemotherapy | 5 | 62.50 |
|  |  |  |  |  |  | No Further Treatment | 2 | 25.00 |
|  |  |  |  |  |  | Other | 1 | 12.50 |
|  |  |  | ICI Monotherapy | 2 | 6.67 | No Further Treatment | 2 | 100.00 |
|  |  |  | Platinum-Based Chemotherapy | 1 | 3.33 | No Further Treatment | 1 | 100.00 |
| Other | 11 | 2.36 | ICI Monotherapy | 3 | 27.27 | No Further Treatment | 1 | 33.33 |
|  |  |  |  |  |  | Non-Platinum-Based Chemotherapy | 1 | 33.33 |
|  |  |  |  |  |  | Platinum-Based Chemotherapy | 1 | 33.33 |
|  |  |  | No Further Treatment | 3 | 27.27 | No Further Treatment | 3 | 100.00 |
|  |  |  | Platinum-Based Chemotherapy | 2 | 18.18 | ICI Monotherapy | 1 | 50.00 |
|  |  |  |  |  |  | No Further Treatment | 1 | 50.00 |
|  |  |  | ICI + Platinum-Based Chemotherapy | 1 | 9.09 | No Further Treatment | 1 | 100.00 |
|  |  |  | ICI – ICI Combination Therapy | 1 | 9.09 | No Further Treatment | 1 | 100.00 |
|  |  |  | Non-Platinum-Based Chemotherapy | 1 | 9.09 | No Further Treatment | 1 | 100.00 |
| ICI + Other Chemotherapy | 7 | 1.50 | No Further Treatment | 4 | 57.14 | No Further Treatment | 4 | 100.00 |
|  |  |  | ICI Monotherapy | 2 | 28.57 | No Further Treatment | 2 | 100.00 |
|  |  |  | Non-Platinum-Based Chemotherapy | 1 | 14.29 | No Further Treatment | 1 | 100.00 |

**Abbreviations:** 1L, first-line; 2L, second-line; 3L, third-line; ES-SCLC, extensive-stage small cell lung cancer; ICI, immune checkpoint inhibitor.

**Supplementary Table 2.** TNM details for ES-SCLC patients.

| Analysis variable | Overall ES-SCLC (n=3484) | De novo ES-SCLC (n=3018) | Progressive/recurrent ES-SCLC (n=466) |
| --- | --- | --- | --- |
| TNM stage value – primary tumor (T) at initial diagnosis |  |  |  |
| T0 | 9 (0.3) | 6 (0.2) | 3 (0.6) |
| T1a | 15 (0.4) | 12 (0.4) | 3 (0.6) |
| T1b | 93 (2.7) | 66 (2.2) | 27 (5.8) |
| T1c | 137 (3.9) | 106 (3.5) | 31 (6.7) |
| T1mi | 6 (0.2) | 6 (0.2) | 0 (0.0) |
| T2a | 197 (5.7) | 157 (5.2) | 40 (8.6) |
| T2b | 180 (5.2) | 144 (4.8) | 36 (7.7) |
| T3 | 280 (8.0) | 238 (7.9) | 42 (9.0) |
| T4 | 362 (10.4) | 317 (10.5) | 45 (9.7) |
| TX | 139 (4.0) | 136 (4.5) | 3 (0.6) |
| Tis | <3 | <3 | <3 |
| Not documented | 2064 (59.2) | 1829 (60.6) | 235 (50.4) |
| TNM stage value – regional lymph nodes (N) at initial diagnosis |  |  |  |
| N0 | 116 (3.3) | 71 (2.4) | 45 (9.7) |
| N1 | 151 (4.3) | 110 (3.6) | 41 (8.8) |
| N2 | 652 (18.7) | 535 (17.7) | 117 (25.1) |
| N3 | 446 (12.8) | 402 (13.3) | 44 (9.4) |
| NX | 118 (3.4) | 115 (3.8) | 3 (0.6) |
| Not documented | 2001 (57.4) | 1785 (59.1) | 216 (46.4) |
| TNM stage value – distant metastasis (M) at initial diagnosis |  |  |  |
| M0 | 297 (8.5) | 77 (2.6) | 220 (47.2) |
| M1 | 375 (10.8) | 370 (12.3) | 5 (1.1) |
| M1a | 132 (3.8) | 128 (4.2) | 4 (0.9) |
| M1b | 133 (3.8) | 132 (4.4) | <3 |
| M1c | 692 (19.9) | 688 (22.8) | 4 (0.9) |
| MX | <3 | <3 | <3 |
| Not documented | 1853 (53.2) | 1622 (53.7) | 231 (49.6) |
